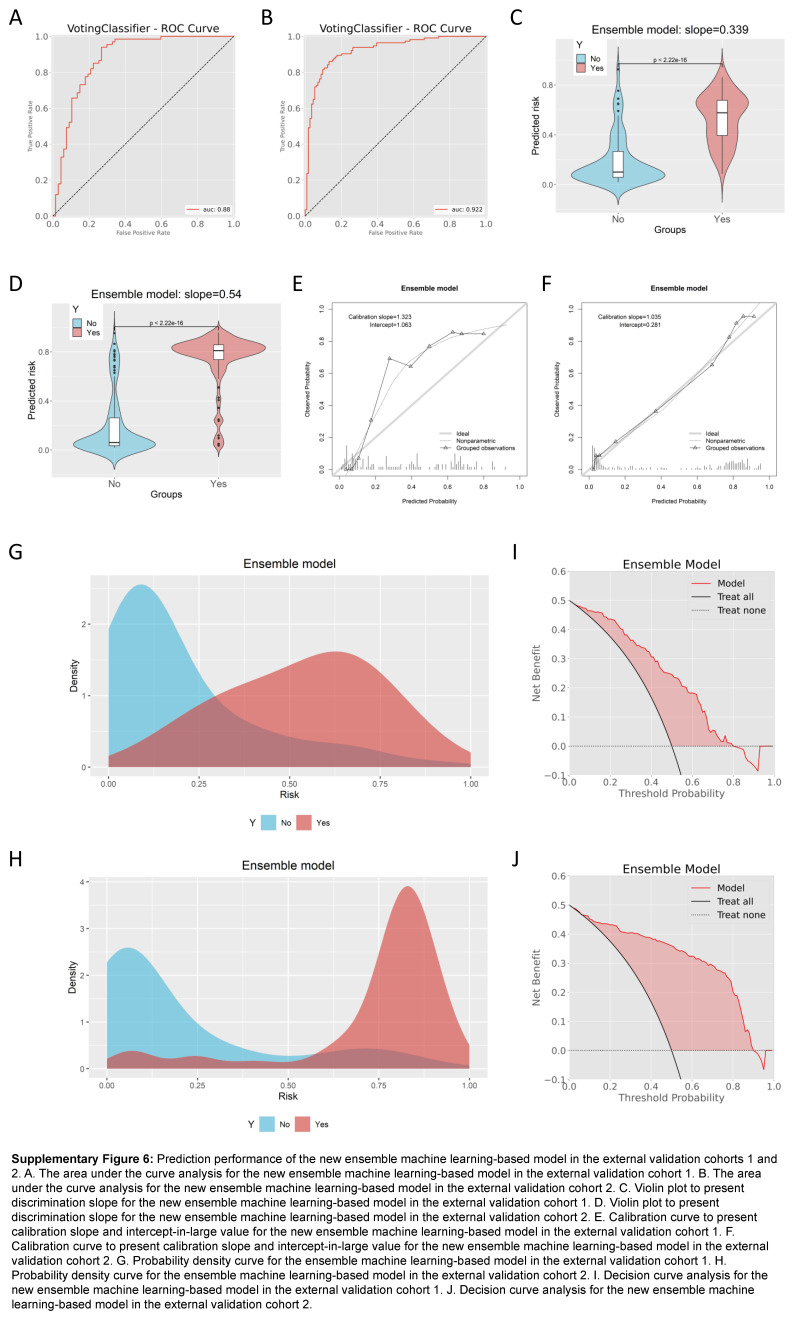# Establishment and validation of an interactive artificial intelligence platform to predict postoperative ambulatory status for patients with metastatic spinal disease: a multicenter analysis

**DOI:** 10.1097/JS9.0000000000001169

**Published:** 2024-02-19

**Authors:** Yunpeng Cui, Xuedong Shi, Yong Qin, Qiwei Wang, Xuyong Cao, Xiaotong Che, Yuanxing Pan, Bing Wang, Mingxing Lei, Yaosheng Liu

**Affiliations:** a Department of Orthopedic Surgery, Peking University First Hospital; b National Clinical Research Center for Orthopedics, Sports Medicine and Rehabilitation; c Department of Orthopedic Surgery, The Fifth Medical Center of PLA General Hospital; d Senior Department of Orthopedics, The Fourth Medical Center of PLA General Hospital; e Department of Orthopedic Surgery, Chinese PLA General Hospital, Beijing; f Department of Orthopedic Surgery, Hainan Hospital of Chinese PLA General Hospital, Sanya; g Department of Evaluation Office, Hainan Cancer Hospital, Haikou; h Department of Joint and Sports Medicine Surgery, The Second Affiliated Hospital of Harbin Medical University, Harbin, People’s Republic of China

**Keywords:** artificial intelligence, feature importance, machine learning, metastatic spinal disease, postoperative ambulatory status

## Abstract

**Background:**

Identification of patients with high-risk of experiencing inability to walk after surgery is important for surgeons to make therapeutic strategies for patients with metastatic spinal disease. However, there is a lack of clinical tool to assess postoperative ambulatory status for those patients. The emergence of artificial intelligence (AI) brings a promising opportunity to develop accurate prediction models.

**Methods:**

This study collected 455 patients with metastatic spinal disease who underwent posterior decompressive surgery at three tertiary medical institutions. Of these, 220 patients were collected from one medical institution to form the model derivation cohort, while 89 and 146 patients were collected from two other medical institutions to form the external validation cohorts 1 and 2, respectively. Patients in the model derivation cohort were used to develop and internally validate models. To establish the interactive AI platform, machine learning techniques were used to develop prediction models, including logistic regression (LR), decision tree (DT), random forest (RF), extreme gradient boosting machine (eXGBM), support vector machine (SVM), and neural network (NN). Furthermore, to enhance the resilience of the study’s model, an ensemble machine learning approach was employed using a soft-voting method by combining the results of the above six algorithms. A scoring system incorporating 10 evaluation metrics was used to comprehensively assess the prediction performance of the developed models. The scoring system had a total score of 0 to 60, with higher scores denoting better prediction performance. An interactive AI platform was further deployed via Streamlit. The prediction performance was compared between medical experts and the AI platform in assessing the risk of experiencing postoperative inability to walk among patients with metastatic spinal disease.

**Results:**

Among all developed models, the ensemble model outperformed the six other models with the highest score of 57, followed by the eXGBM model (54), SVM model (50), and NN model (50). The ensemble model had the best performance in accuracy and calibration slope, and the second-best performance in precise, recall, specificity, area under the curve (AUC), Brier score, and log loss. The scores of the LR model, RF model, and DT model were 39, 46, and 26, respectively. External validation demonstrated that the ensemble model had an AUC value of 0.873 (95% CI: 0.809–0.936) in the external validation cohort 1 and 0.924 (95% CI: 0.890–0.959) in the external validation cohort 2. In the new ensemble machine learning model excluding the feature of the number of comorbidities, the AUC value was still as high as 0.916 (95% CI: 0.863–0.969). In addition, the AUC values of the new model were 0.880 (95% CI: 0.819–0.940) in the external validation cohort 1 and 0.922 (95% CI: 0.887–0.958) in the external validation cohort 2, indicating favorable generalization of the model. The interactive AI platform was further deployed online based on the final machine learning model, and it was available at https://postoperativeambulatory-izpdr6gsxxwhitr8fubutd.streamlit.app/. By using the AI platform, researchers were able to obtain the individual predicted risk of postoperative inability to walk, gain insights into the key factors influencing the outcome, and find the stratified therapeutic recommendations. The AUC value obtained from the AI platform was significantly higher than the average AUC value achieved by the medical experts (*P*<0.001), denoting that the AI platform obviously outperformed the individual medical experts.

**Conclusions:**

The study successfully develops and validates an interactive AI platform for evaluating the risk of postoperative loss of ambulatory ability in patients with metastatic spinal disease. This AI platform has the potential to serve as a valuable model for guiding healthcare professionals in implementing surgical plans and ultimately enhancing patient outcomes.

## Introduction

Highlights

- The interactive AI platform can accurately predict postoperative ambulatory status.
- The ensemble model obtained the highest score in a comprehensive scoring system.
- External validation confirms high predictive capability of the AI model.
- The AI platform outperforms medical experts in predicting risk.

Metastatic spinal disease is a common complication of cancer, with a reported incidence of 70%^1,2^. It poses significant challenges for patients and clinicians due to its detrimental effects on neurological function and quality of life^3,4^. The disease is characterized by the spread of cancer cells to the vertebral column, leading to spinal instability, compression of neural elements, pain, and neurological deficits^4,5^. The management of metastatic spinal disease often involves surgical intervention to relieve pain, decompress neural structures, and restore spinal stability^6–8^. Notably, the ability to maintain or regain ambulatory status after surgery is a critical factor in determining the success of the procedure and the overall prognosis for patients^9^.

Despite the importance of postoperative ambulatory status, there is a lack of robust clinical tools to predict whether a patient will have the ability to walk after surgery among patients with metastatic spinal disease. Currently, the literature on this topic is limited, with only a few studies reporting on specific risk factors associated with postoperative ambulatory status^10–14^, and some traditional scoring system being developed based on survival prediction to stratify function outcomes^8,15,16^. As a result, surgeons lack reliable guidance to inform their postoperative functional outcome for patients with metastatic spinal disease^17^. Therefore, there is a pressing need for the development of accurate prediction models to assess postoperative ambulatory status in these patients.

In recent years, the application of artificial intelligence (AI) and machine learning techniques in the field of spinal metastatic tumors has demonstrated excellent achievements^18–20^. Machine learning algorithms can effectively analyze complex datasets and identify patterns and relationships that may not be readily apparent to human observers. These algorithms have the potential to improve the accuracy and precision of prediction models^19^, providing valuable insights into the prognosis and outcomes of patients with metastatic spinal disease^21,22^.

Therefore, the objective of this study is to establish and validate an AI platform for predicting postoperative ambulatory status in patients with metastatic spinal disease. Furthermore, we will conduct a comparative analysis between the predictions made by spine surgeons and the AI platform to assess its performance in clinical practice. The development of this AI platform has the potential to assist clinicians in making informed decisions regarding surgical intervention and improving patient outcomes.

## Methods

### Patients and study design

**Figure 1:**
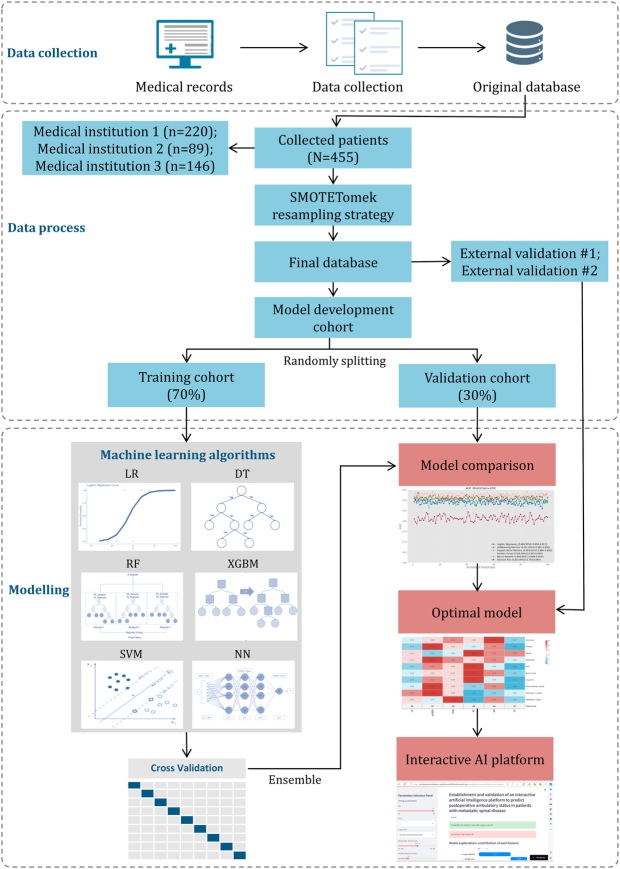
Schematic depiction of study design and machine learning process.

This study collected 455 patients with metastatic spinal disease who underwent posterior decompressive surgery at three tertiary medical institutions from January 2015 to May 2023. Of these, 220 patients were prospectively collected from one medical institution to form the model derivation cohort, while 89 and 146 patients were collected from two other medical institutions to form the external validation cohorts 1 and 2, respectively. The three medical institutions in this study, two of which are located in the northern region of our country and one in the southern region, are all teaching hospitals and are classified as tertiary A-grade hospitals with good reputations. Validating the model in different regions can further confirm its effectiveness by demonstrating its ability to perform consistently across diverse populations and healthcare settings. All patients underwent X-ray and MRI scans to confirm the location of the metastatic lesion. The study included patients who met specific criteria, including the presence of radiographic evidence of metastatic spinal disease and at least one of the following symptoms: progressive local mechanical or radiation pain, impairment of sensory function, lower limb motor function, or sphincter function. Patients who were receiving conservative treatment, those with primary spinal tumors, metastatic spinal disease caused by leukemia, and intramedullary metastases of spinal metastases were excluded from the study. Additionally, patients who had previously undergone surgery at the site of spinal metastases were also excluded. A flowchart illustrating the study design is presented in Figure 1. Patients in the model derivation cohort were randomly divided into a training cohort and an internal validation cohort using a 7:3 ratio. Patients from external validation cohorts 1 and 2 were utilized for the external validation of the model. Based on the specified inclusion and exclusion criteria and the objectives of the analysis, the study employed a per-protocol analysis method, and the study protocol is provided in Supplementary File 1 (Supplemental Digital Content 1, http://links.lww.com/JS9/B973). The study protocol was approved by the research ethics board of our institution, and all patients provided informed consent for the review of their medical records and images. This study was registered at a National Clinical Trial Registry Center. The study was conducted in accordance with the guidelines outlined in the Declaration of Helsinki, and the reporting of the study adhered to the strengthening the reporting of cohort, cross-sectional, and case–control studies in surgery (STROCSS) criteria^23^ and the TRIPOD Checklist^24^ (Supplemental Digital Content 2, http://links.lww.com/JS9/B974).

### Surgical process

The decision to perform surgery was based on indications such as intractable pain due to spinal instability and myelopathy caused by spinal cord compression. The appropriate surgical approach was determined through multidisciplinary collaboration among a neuro-radiologist, spinal tumor surgeon, and oncologist. The surgical management of spinal metastases involved a complex procedure comprising palliative decompression, partial vertebrectomy/*En bloc* resection of vertebrae, and internal fixation using pedicle screw instrumentation. Under general anesthesia, the patient was positioned in the prone position on the operating table. A midline skin incision was made over the affected area of the spine, and the muscles were carefully dissected to expose the posterior elements of the spine. The surgical procedure involved a posterior approach with either laminectomy or laminotomy to gain access to the spinal cord and nerve roots. The tumor was then meticulously removed using a combination of vertebrectomy and tumor debulking techniques. The extent of the vertebrectomy depended on the tumor involvement, and the decision to perform either subtotal or total vertebrectomy was based on the surgeon’s assessment of the tumor and the patient’s overall health status. No intradural work was required in the present study. Following tumor excision, the resultant space within the vertebral body was filled with bone cement and artificial vertebral bodies to facilitate fusion and stabilization of the spine. Fusion was achieved by addressing the adjacent vertebrae above and below the corpectomy site, involving the insertion of screws into the pedicles of the affected vertebrae, which were interconnected using rods to ensure stability and mitigate the risk of further deformity. The pedicle screw instrumentation also facilitated the maintenance of the correction achieved during the vertebrectomy. The wound was closed in layers using sutures.

### Evaluation of the primary outcome

The primary outcome of this study was the ambulatory status of the patients within one week after surgery. Ambulatory status was defined as the ability to independently take at least two steps with each foot (totaling four steps), even if the use of a cane or walker was necessary^6^. The ability to ambulate after surgery for metastatic spinal disease is of great importance as it directly impacts the patients’ overall quality of life and functional independence^25^.

### Quality control

In this study, a meticulous approach was adopted to safeguard the precision and dependability of the acquired data. To begin with, extensive training was provided to the research team, equipping them with a thorough understanding of the data collection protocols. The primary objective of this training was to minimize potential errors and ensure uniform adherence to standardized guidelines by all team members during the data collection and recording processes. Subsequently, a rigorous data entry and validation framework was implemented, involving a meticulous double-entry verification method where two independent individuals entered the data, followed by a meticulous cross-verification process to detect any disparities. Additionally, comprehensive data validation checks were performed to meticulously identify and rectify any inconsistencies or inaccuracies within the collected data.

Moreover, an exhaustive data cleaning procedure was meticulously executed to detect and rectify errors, missing data points, or outliers. This involved a meticulous comparison of the collected data with the source documents, diligently resolving any disparities uncovered. Furthermore, continuous data monitoring was conducted throughout the study, facilitating the proactive identification of potential issues or trends that could impact data quality. This encompassed periodic audits, meticulous review of data collection forms, and the provision of constructive feedback to the research team to ensure ongoing data quality control. By adhering to these stringent data quality control measures, the study aimed to uphold a standard of excellence in data quality, thereby fortifying the validity and integrity of the research findings.

### Data preparation

Data preprocessing pipeline was utilized to ensure consistent and reproducible transformation of the data using scikit-learn. The pipeline combined multiple preprocessing steps into a single object for improved accuracy and reliability of machine learning models. It involved data transformation, feature selection, data splitting, and standardization and normalization. Imbalanced data was addressed using a SMOTETomek resampling strategy, which combines Synthetic Minority Oversampling Technique (SMOTE) and Tomek Links Undersampling^26^. This strategy generates a new dataset with a larger sample size and a more balanced distribution of data, enhancing statistical power and generalizability of the findings. For example, the sample size of the model derivation cohort increased to 334 after implementing the SMOTETomek resampling strategy, with a positive rate of the primary outcome at 50%. Similarly, cohorts 1 and 2 also saw an increase in sample size to 134 and 228, respectively. In addition, a stratified strategy was employed to maintain consistent proportions of the outcome classes between ambulatory and nonambulatory patients.

### Modeling

A comprehensive analysis was conducted using a range of models, including logistic regression (LR) and five machine learning algorithms: extreme gradient boosting machine (eXGBM), support vector machine (SVM), random forest (RF), neural network (NN), and decision tree (DT). Furthermore, to enhance the resilience of the study’s model, an ensemble machine learning approach was employed using a soft-voting method^27,28^. All models were provided with the same input features to ensure consistency, and the model features were identify using subgroup analysis. Grid and random hyperparameter searches were performed to identify the optimal hyperparameters for each model, with the area under the curve (AUC) used as the optimization metric. During the grid and random hyperparameter searches to identify the optimal hyperparameters for each model, the model parameters were optimized and validated through 10-fold cross-validation on the training data. By training and evaluating the model on different combinations of hyperparameters using 10-fold cross-validation, we obtained a more reliable estimate of the model’s performance. In addition, to accommodate the variability in model performance, wide ranges were established for the hyperparameters. For instance, the DT depth range was set from 2 to 100, allowing for a broad exploration of different tree depths. Similarly, the ‘min_child_weight’ hyperparameter was varied from 1 to 100, enabling the model to consider a wide range of weights for the minimum number of samples required to create a new child node. The ‘min_samples_split’ and ‘min_samples_leaf’ hyperparameters were set from 2 to 200, allowing for flexibility in determining the minimum number of samples needed to split an internal node or form a leaf node, respectively. Learning curves were used as a tool in the identification of overfitting and underfitting issues in models. In the learning curve, the model’s performance on both the training and validation datasets was plotted against the number of training instances or iterations. Machine learning algorithms were implemented using Python (version 3.9.7), and hyperparameter tuning was conducted using Python scikit-learn (version 1.2.2).

### Validation

The internal and external validation cohorts were used to validate the models, and multiple evaluation metrics were employed, including the AUC, accuracy, precision, recall, specificity, Brier score, log loss, discrimination slope, calibration slope, and intercept-in-large value. The AUC value was obtained using 100 bootstraps. The accuracy, precision, and recall were calculated using a confusion matrix. In the following equation, TP, TN, FP, and FN represent true positive, true negative, false positive, and false negative, respectively.


Accuracy=(TP+TN)/(TP+FN+FP+TN)

Precision=TP/(TP+FP)

Recall(Sensitivity)=TP/(TP+FN)

Specificity=1−FP/(TN+FP)

The Brier score, was calculated using the formula where N represents the total sample, $p_{i}$ represents the predicted risk, and $o_{i}$ represents the actual probability.


$$\mathrm{Brier}\;\mathrm{Score}=\frac{1}{N}\sum_{i=1}^{n}{\left(p_{i}-o_{i}\right)}^{2}\;$$

The log loss, calculated using the scikit-learn formula, is a metric that evaluates the quality of classification model predictions. It takes into account the number of samples ( N ), the number of classes ( M ), the true labels ( $y_{\mathrm{ij}}$ ), and the predicted probabilities ( $p_{\mathrm{ij}}$ ).


$$\mathrm{Log}\;\mathrm{Loss}=-\frac{1}{N}\sum_{i=1}^{N}\sum_{j=1}^{M}y_{\mathrm{ij}}\;\log(p_{\mathrm{ij}})\;$$

The discrimination slope was calculated as the mean difference between the predicted probabilities of patients with and without postoperative ambulatory status. The calibration slope and intercept-in-large value were obtained from the calibration curve. Additionally, a scoring system was used to comprehensively evaluate the prediction performance of the models^29,30^, with each metric rated on a scale of 1 to 6. The scoring system ranged from 0 to 60. Finally, decision curve analysis (DCA) was employed to determine the clinical net benefits for each model.

### Feature importance

The Shapley additive explanation (SHAP) values were used to determine the importance of model features^26^. The SHAP values were calculated using the equation, where g represents the interpretation model, M represents the number of input parameters, $\phi_{0}$ represents a constant, $\phi_{j}$ represents the Shapley value of each model feature, and ${Z^{\prime }}_{j}$ represents the coalition vector.


$$g\left(z^{\prime }\right)=\phi_{0}+\sum_{j=1}^{M}\phi_{j}{Z^{\prime }}_{j}$$

Among the coalition vectors, ‘1’ suggests that the feature is the same as the feature of the case x to be explained, while ‘0’ suggests that the feature is missing in the present case x . Therefore, considering case x as all simplified features were 1, and then the SHAP expression could be simplified and outlined below.


$$g\left(x^{\prime }\right)=\phi_{0}+\sum_{j=1}^{M}\phi_{j}$$

### Establishment of the interactive AI platform

An AI platform was developed to estimate the risk of not gaining ambulatory status in patients undergoing decompressive surgery for metastatic spinal disease. The AI platform was designed to be user-friendly and accessible using the Streamlit. The code of supporting the development of the AI platform is available at https://github.com/Starxueshu/postoperativeambulatory. It allows users to customize the input parameters, calculates the probability of not gaining ambulatory status based on the selected parameters, and provides an interface that explains the model’s methodology and performance. Patients were categorized into high-risk or low-risk groups based on a threshold, and corresponding intervention recommendations were provided in terms of the patient stratification. In addition, a human-machine comparative experiment was conducted to compare the prediction performance of 6 medical experts and the AI platform in predicting the outcome of not gaining ambulatory status among patients with spinal metastatic tumors. AUC values were calculated for each medical expert.

### Statistical analysis

Continuous variables were summarized using the mean and SD for normally distributed data, while median and interquartile range (IQR) were utilized for non-normally distributed data. Categorical variables were presented as proportions. Student’s *t*-test was used to compare normally distributed continuous variables, while Wilcoxon Rank Sum Test (Mann–Whitney *U* test) was used to compare non-normally distributed variables. The *χ* ^2^ test was conducted to compare the distribution of categorical variables. Statistical power analysis was conducted for significant variables. Delong test was used to compare the prediction performance between medical experts and the AI platform. Statistical analysis was performed using the R language program (version 4.1.2), and a *P*-value less than 0.05 was considered statistically significant with two-sided testing.

## Results

### Patient clinical characteristics

In the model derivation cohort, 220 patients were collected for analysis in the study. The median age was 60.00 (53.00, 68.00) years, with 67.3% of patients being male and 20.9% of patients being current smokers (Table 1). The most common primary tumor was rapid growth (38.6%), followed by moderate growth (35.0%) and slow growth (26.4%). The burden of comorbidities was relatively heavy, since 47.3% of patients had at least one comorbidity. In detail, the most prevalent comorbidities were hypertension (30.91%) and diabetes (13.64%) (Supplementary Table 1, Supplemental Digital Content 3, http://links.lww.com/JS9/B975). Regarding systematic therapies, preoperative chemotherapy, targeted therapy, and endocrinology therapy accounted for 17.3, 9.5, and 9.1%, respectively. The tumor burden was relatively heavy, because there were 43.6% of patients had extravertebral bone metastases, 26.8% of patients had visceral metastases, and 19.1% of patients had an Eastern Cooperative Oncology Group (ECOG) score of four, indicating being unable to take care of oneself in daily life. The majority of patients were treated with palliative decompression (89.5%) at thoracic and thoracolumbar site (67.7%). In the entire cohort, 58.6% of patients had the Bilsky score of 3, indicating severe spinal cord compression, and 49.1% of patients lost their ability to walk before surgery. During surgery, 80.5 of patients received intraoperative blood transfusion. More detailed information on preoperative laboratory examination, including albumin, cholesterol, hemoglobin, and PT, is summarized in Table 1.

**Table 1:** Patient’s clinical characteristics and a comparison of clinical characteristics between patients with and without postoperative walking ability.

		*Postoperative ambulatory status*		
Characteristics	Overall	Yes	No	*P*
*n*	220	169	51	
Age (years, median [IQR])	60.00 [53.00–68.00]	60.00 [53.00–67.00]	63.00 [56.00–74.00]	0.041
Sex (male/female, %)	148/72 (67.3/32.7)	110/59 (65.1/34.9)	38/13 (74.5/25.5)	0.277
Primary tumor (%)				0.557
Slow growth	58 (26.4)	46 (27.2)	12 (23.5)	
Moderate growth	77 (35.0)	61 (36.1)	16 (31.4)	
Rapid growth	85 (38.6)	62 (36.7)	23 (45.1)	
Tumor type (%)				0.254
Thyroid cancer	5 (2.3)	5 (3.0)	0 (0.0)	
Prostate cancer	31 (14.1)	23 (13.6)	8 (15.7)	
Breast cancer	20 (9.1)	17 (10.1)	3 (5.9)	
Renal cancer	43 (19.5)	35 (20.7)	8 (15.7)	
Lung cancer	58 (26.4)	41 (24.3)	17 (33.3)	
Hepatocellular carcinoma	13 (5.9)	9 (5.3)	4 (7.8)	
Gastrointestinal system cancer	12 (5.5)	11 (6.5)	1 (2.0)	
Urogenital cancer	8 (3.6)	8 (4.7)	0 (0.0)	
Others	30 (13.6)	20 (11.8)	10 (19.6)	
Smoking (%)				0.773
Never	162 (73.6)	125 (74.0)	37 (72.5)	
Previous	12 (5.5)	10 (5.9)	2 (3.9)	
Current	46 (20.9)	34 (20.1)	12 (23.5)	
BMI (kg/m^2^, median [IQR])	23.90 [21.34–26.12]	23.66 [21.23–26.12]	24.03 [22.04–26.20]	0.419
Number of comorbidities (%)				0.050
0	116 (52.7)	93 (55.0)	23 (45.1)	
1	68 (30.9)	54 (32.0)	14 (27.5)	
≧2	36 (16.4)	22 (13.0)	14 (27.5)	
Coronary disease (no/yes, %)	211/9 (95.9/4.1)	164/5 (97.0/3.0)	47/4 (92.2/7.8)	0.254
Diabetes (no/yes, %)	191/29 (86.8/13.2)	151/18 (89.3/10.7)	40/11 (78.4/21.6)	0.074
Hypertension (no/yes, %)	150/70 (68.2/31.8)	121/48 (71.6/28.4)	29/22 (56.9/43.1)	0.071
Preoperative chemotherapy (no/yes, %)	182/38 (82.7/17.3)	140/29 (82.8/17.2)	42/9 (82.4/17.6)	1.000
Preoperative targeted therapy (no/yes, %)	199/21 (90.5/9.5)	153/16 (90.5/9.5)	46/5 (90.2/9.8)	1.000
Preoperative endocrinology (no/yes, %)	200/20 (90.9/9.1)	157/12 (92.9/7.1)	43/8 (84.3/15.7)	0.112
Extravertebral bone metastasis (no/yes, %)	124/96 (56.4/43.6)	100/69 (59.2/40.8)	24/27 (47.1/52.9)	0.171
Viscera metastases (no/yes, %)	161/59 (73.2/26.8)	119/50 (70.4/29.6)	42/9 (82.4/17.6)	0.132
ECOG (%)				<0.001
1	3 (1.4)	3 (1.8)	0 (0.0)	
2	108 (49.1)	106 (62.7)	2 (3.9)	
3	67 (30.5)	51 (30.2)	16 (31.4)	
4	42 (19.1)	9 (5.3)	33 (64.7)	
Surgical process (%)				0.069
Palliative decompression	197 (89.5)	147 (87.0)	50 (98.0)	
Partial resection of vertebrae	12 (5.5)	11 (6.5)	1 (2.0)	
En bloc resection of vertebrae	11 (5.0)	11 (6.5)	0 (0.0)	
Surgical site (%)				0.011
Cervical and cervical thoracic	9 (4.1)	7 (4.1)	2 (3.9)	
Thoracic and thoracolumbar	149 (67.7)	106 (62.7)	43 (84.3)	
Lumbar and lumbosacral	62 (28.2)	56 (33.1)	6 (11.8)	
Number of surgical segments (%)				0.237
1	108 (49.1)	88 (52.1)	20 (39.2)	
2	68 (30.9)	48 (28.4)	20 (39.2)	
≥3	44 (20.0)	33 (19.5)	11 (21.6)	
Preoperative albumin (g/l, median [IQR])	40.10 [37.20–42.60]	40.60 [37.50–43.20]	38.90 [36.75–41.50]	0.034
Total cholesterol (mmol/l, median [IQR])	4.42 [3.71–5.10]	4.58 [3.83–5.19]	4.08 [3.50–4.78]	0.004
Preoperative hemoglobin (g/l, median [IQR])	133.00 [119.00–143.00]	133.00 [124.00–144.00]	131.00 [112.00–141.50]	0.188
PT (seconds, median [IQR])	11.29 [10.60–11.90]	11.20 [10.50–11.70]	11.80 [11.10–12.50]	0.001
Bilsky score (%)				<0.001
1	28 (12.7)	27 (16.0)	1 (2.0)	
2	63 (28.6)	58 (34.3)	5 (9.8)	
3	129 (58.6)	84 (49.7)	45 (88.2)	
Preoperative ambulatory status (yes/no, %)	112/108 (50.9/49.1)	110/59 (65.1/34.9)	2/49 (3.9/96.1)	<0.001
Intraoperative blood transfusion (ml, %)				0.799
None	43 (19.5)	33 (19.5)	10 (19.6)	
<1000	122 (55.5)	92 (54.4)	30 (58.8)	
≧1000	55 (25.0)	44 (26.0)	11 (21.6)	

ECOG, Eastern cooperative oncology group; IQR, Interquartile range; PT, Prothrombin time.

### Identification of risk factors by subgroup analysis

Subgroup analysis revealed that patients who experienced postoperative inability to walk exhibited certain characteristics. They tended to be older (*P*=0.041), have a higher number of comorbidities (*P*=0.050), have a higher ECOG score (*P*<0.001), a higher rate of surgical site involvement in the thoracic and thoracolumbar regions (*P*=0.011), lower levels of preoperative albumin (*P*=0.034) and total cholesterol (*P*=0.004), higher levels of prothrombin time (PT) (*P*=0.001), higher degrees of Bilsky score (*P*<0.001), and a higher rate of preoperative inability to walk (*P*<0.001) (Table 1). Consequently, these nine variables were utilized as feature inputs to train and optimize machine learning-based models.

### Modeling and prediction evaluation

**Figure 2:**
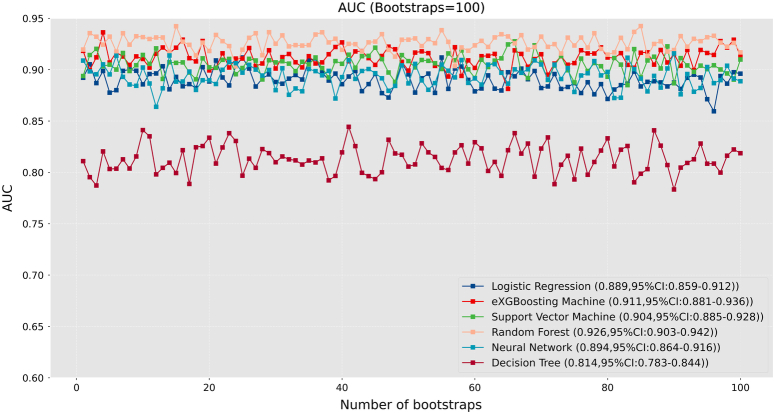
Area under the curve analysis for machine learning-based models.

**Figure 3:**
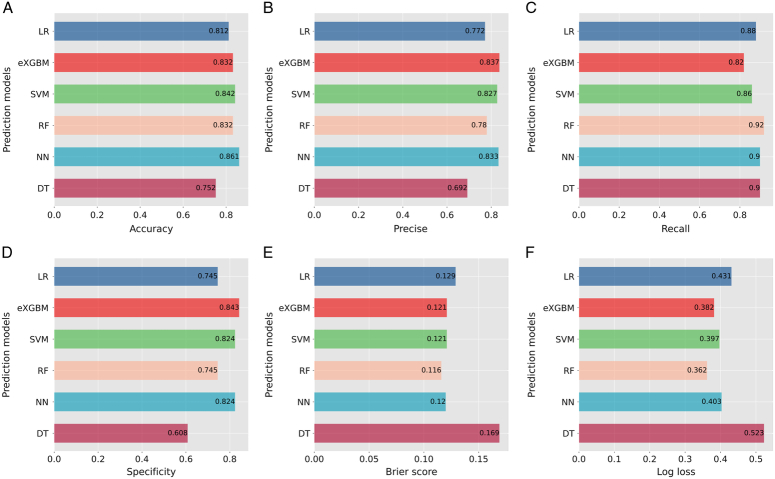
Evaluation of prediction performance for machine learning-based models. A. Accuracy; B. Precise; C. Recall; D. Specificity; E. Brier score; F. Log loss. DT, decision tree; eXGBM, extreme gradient boosting machine; LR, logistic regression; NN, neural network; RF, random forest; SVM, support vector machine.

**Figure 4:**
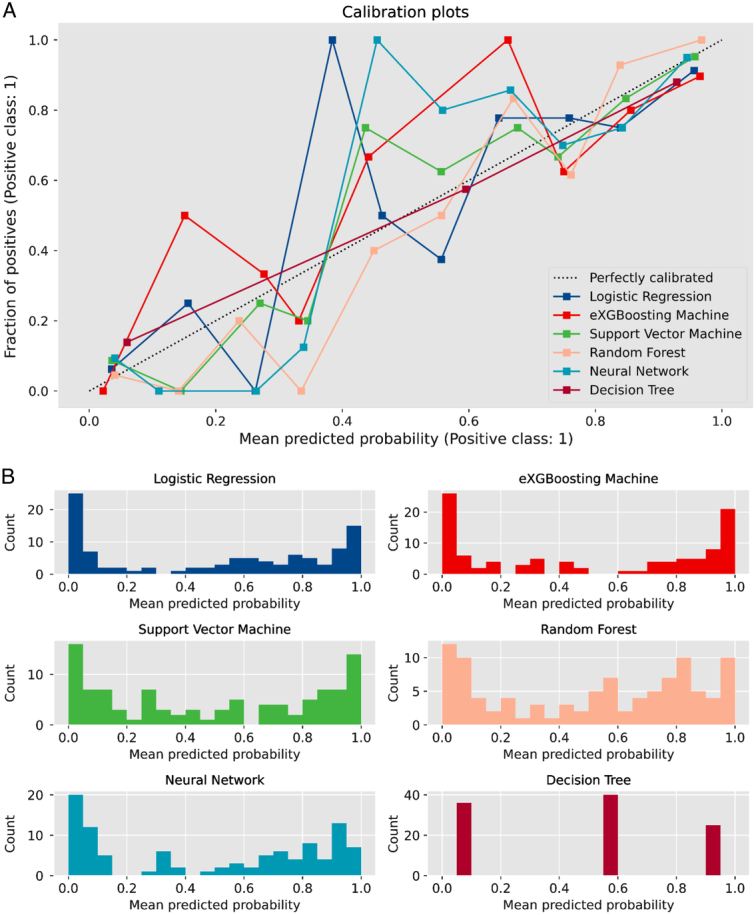
Calibration curve and histogram of predicted probability for machine learning-based models. A. Calibration curve for machine learning-based models. In the curve, the *x*-axis represents the predicted probabilities or scores generated by the models, while the *y*-axis represents the observed probabilities or actual outcomes. The curve visually displays how well the model’s predictions align with the actual outcomes. A perfectly calibrated model would exhibit a diagonal line, indicating a close match between predicted and observed probabilities; B. Histogram of predicted probability of postoperative inability to walk for machine learning-based models.

**Figure 5:**
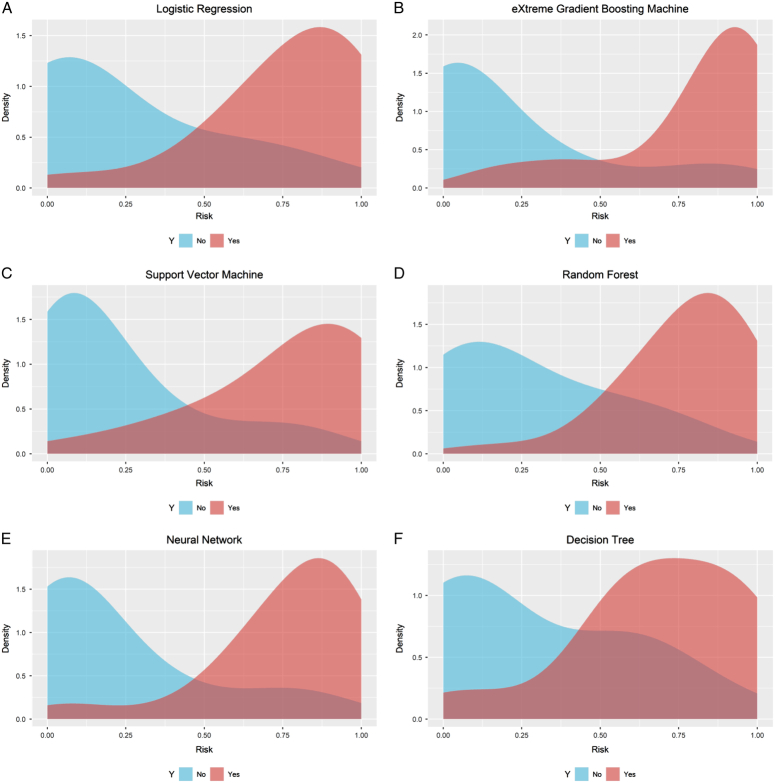
Probability density curve for machine learning-based models. A. Logistic regression; B. Extreme gradient boosting machine; C. Support vector machine; D. Random forest; E. Neural network; F. Decision tree. The curve was plotted with predicted risk against probability density. The blue indicates patients who were able to walk after surgery, whereas the red indicates patients who were unable to walk after surgery.

**Figure 6:**
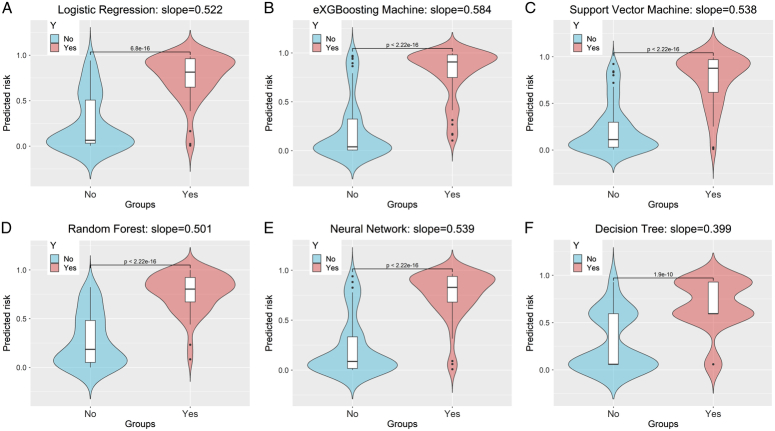
Violin plot to present discrimination slope value for machine learning-based models. A. Logistic regression; B. Extreme gradient boosting machine; C. Support vector machine; D. Random forest; E. Neural network; F. Decision tree. The blue indicates patients who were able to walk after surgery, whereas the red indicates patients who were unable to walk after surgery.

**Figure 7:**
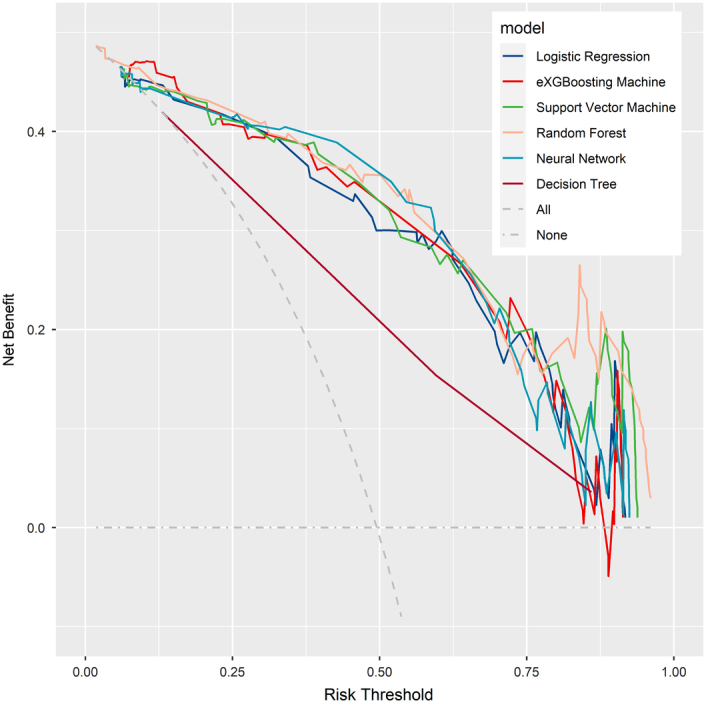
Decision curve analysis for machine learning-based models. The *x*-axis represents the threshold probability, which is the probability at which a positive outcome is considered. The *y*-axis represents the net benefit, which is the difference between the proportion of true positives and the proportion of false positives, adjusted for the relative harm of false positives and false negatives. The curve visually demonstrates the net benefit of using the models across different threshold probabilities. The higher the curve lies above the baseline, the greater the potential benefit of using the models in decision-making.

Before training the models, the SMOTETomek resampling strategy was conducted to produce a new database with more balanced data distribution, and the incidences of the positive and negative outcome were both 50%. The baseline clinical characteristics of the new database are presented in Supplementary Table 2 (Supplemental Digital Content 4, http://links.lww.com/JS9/B976). A comparison of these clinical characteristics was performed between the training cohort and the internal validation cohort, and the results indicated that all the clinical characteristics were similar (All *P*>0.05, Supplementary Table 3, Supplemental Digital Content 5, http://links.lww.com/JS9/B977). This suggests that there were no significant differences in the baseline clinical characteristics between the two groups. The hyperparameters of the all models are presented in Supplementary Table 4 (Supplemental Digital Content 6, http://links.lww.com/JS9/B978). Among all the six developed models, the RF model demonstrated the highest AUC value of 0.926 (95% CI: 0.903–0.942), followed by the eXGBM model (0.911, 95% CI: 0.881–0.936) and the SVM model (0.904, 95% CI: 0.885–0.928) (Fig. 2 and Table 2). However, the NN model exhibited the highest accuracy (0.861), followed by the SVM model (0.842), the eXGBM model (0.832), and the RF model (0.832). The eXGBM model demonstrated the best prediction performance in terms of precision (0.837) and specificity (0.843), and the second-best performance in terms of log loss (0.382) (Fig. 3). The calibration curve for each model, depicted in Figure 4A, indicated favorable calibration as the curves were closely aligned with the perfectly calibrated curve. Calculation of the calibration slope and intercept-in-large value for each model revealed that the eXGBM model had the best intercept-in-large value (−0.002), which was the closest to 0, and the SVM model had the best calibration slope (0.859), which was the closest to 1 (Supplementary Figure 1, Supplemental Digital Content 7, http://links.lww.com/JS9/B979). Figure 4B displayed plots of the mean predicted probability against count for each model, revealing distinct distribution patterns of predicted risk of experiencing inability to walk. When the mean predicted probability was classified by the actual postoperative ambulatory status (Fig. 5), density curve analysis demonstrated that, with the exception of the DT model, other models, particularly the eXGBM model and NN model, exhibited excellent discrimination ability. These models displayed less overlap and a larger distinguishing area between ambulatory and nonambulatory patients. The violin plot of discrimination slope confirmed that the eXGBM model had the highest discrimination slope (0.584), followed by the NN model (0.539) and the SVM model (0.538) (Fig. 6). DCA revealed similar trends, with the models exhibiting favorable clinical net benefits, except for the DT model (Fig. 7).

**Table 2:** Prediction performance of models.

	Models						
Metrics	LR	eXGBM	SVM	RF	NN	DT	Ensemble
Accuracy	0.812	0.832	0.842	0.832	0.861	0.752	0.861
Precise	0.772	0.837	0.827	0.780	0.833	0.692	0.833
Recall	0.880	0.820	0.860	0.920	0.900	0.900	0.900
Specificity	0.745	0.843	0.824	0.745	0.824	0.608	0.824
AUC (95% CI)	0.889 (0.859–0.912)	0.911 (0.881–0.936)	0.904 (0.885–0.928)	0.926 (0.903–0.942)	0.894 (0.864–0.916)	0.814 (0.783–0.844)	0.911 (0.854–0.968)
Brier score	0.129	0.121	0.121	0.116	0.120	0.169	0.118
Log loss	0.431	0.382	0.397	0.362	0.403	0.523	0.375
Discrimination slope	0.522	0.584	0.538	0.501	0.539	0.399	0.513
Intercept-in-large value	0.009	−0.002	0.032	−0.304	0.098	0.079	−0.043
Calibration slope	0.771	0.732	0.859	1.337	0.854	0.706	1.086
Total score	39	54	50	46	50	26	57

AUC, area under the curve; DT, decision tree; eXGBM, eXtreme gradient boosting machine; LR, logistic regression; NN, neural network; RF, random forest; SVM, support vector machine.

### Ensemble machine learning-based model

**Figure 8:**
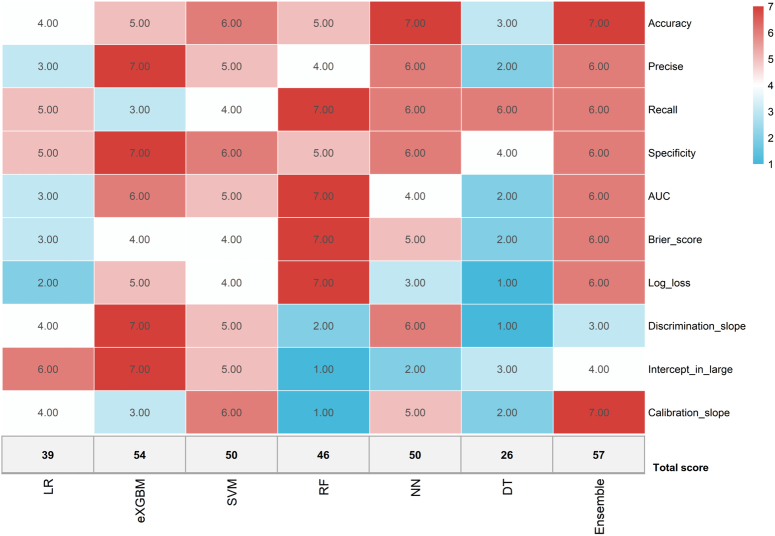
Heatmap to comprehensively evaluate the prediction performance for machine learning-based models and the ensemble model. Red indicates superior prediction performance, while blue indicates relatively inferior prediction performance.

To further improve the prediction performance of machine learning model, an ensemble model was introduced to combine the results of the six algorithms. In this model, the AUC value was 0.911 (95% CI: 0.854–0.968) (Supplementary Figure 2A, Supplemental Digital Content 8, http://links.lww.com/JS9/B980), with the accuracy, precise, recall, and specificity of 0.861, 0.833, 0.900, and 0.824, respectively (Table 2). The discrimination slope was 0.513 (Supplementary Figure 2B, Supplemental Digital Content 8, http://links.lww.com/JS9/B980), and the density curve suggested favorable discrimination of the model (Supplementary Figure 2C, Supplemental Digital Content 8, http://links.lww.com/JS9/B980). DCA demonstrated its clinical usefulness (Supplementary Figure 2D, Supplemental Digital Content 8, http://links.lww.com/JS9/B980). In addition, according to the comprehensive evaluation scoring system, the ensemble model outperformed the other six models with the highest score of 57, followed by the eXGBM model (54), the SVM model (50), and the NN model (50) (Fig. 8). In detail, among the seven models, the ensemble model had the best performance in accuracy and calibration slope (Supplementary Figure 2E, Supplemental Digital Content 8, http://links.lww.com/JS9/B980), and the second-best performance in precise, recall, specificity, AUC, Brier score, and log loss. The scores of the LR model, RF model, and DT model were 39, 46, and 26, respectively. Therefore, the ensemble model was served as the optimal model in this study.

### External validation

External validation was performed on two cohorts (*n*=89 and *n*=146) using the optimal model. The baseline characteristics of the two cohorts can be found in Supplementary Table 5 (Supplemental Digital Content 9, http://links.lww.com/JS9/B981) and Supplementary Table 6 (Supplemental Digital Content 10, http://links.lww.com/JS9/B982), respectively. After conducting the SMOTETomek resampling strategy, we obtained two new and more balanced cohorts, with the incidences of positive and negative outcome both being 50% in the external validation cohort 1 (*n*=134, Supplementary Table 7, Supplemental Digital Content 11, http://links.lww.com/JS9/B983) and external validation cohort 2 (*n*=228, Supplementary Table 8, Supplemental Digital Content 12, http://links.lww.com/JS9/B984). The results demonstrated that the model exhibited favorable predictive performance in the two external validation cohorts. Specifically, the AUC value was 0.873 (95% CI: 0.809–0.936) in the external validation cohort 1 (Supplementary Figure 3A, Supplemental Digital Content 13, http://links.lww.com/JS9/B985) and 0.924 (95% CI: 0.890–0.959) in the external validation cohort 2 (Supplementary Figure 3B, Supplemental Digital Content 13, http://links.lww.com/JS9/B985), indicating a strong discriminative power of the ensemble model in distinguishing between patients who were likely to experience postoperative inability to walk and those who were not. In addition, the accuracy was 0.739 in the external validation cohort 1 and 0.855 in the external validation cohort 2 (Supplementary Table 9, Supplemental Digital Content 14, http://links.lww.com/JS9/B986), with the corresponding precises of 0.864 and 0.846, respectively. The discrimination slopes were 0.316 (Supplementary Figure 3C, Supplemental Digital Content 13, http://links.lww.com/JS9/B985) and 0.533 (Supplementary Figure 3D, Supplemental Digital Content 13, http://links.lww.com/JS9/B985), respectively, and the density curves (Supplementary Figure 3E, Supplemental Digital Content 13, http://links.lww.com/JS9/B985 and Supplementary Figure 3F, Supplemental Digital Content 13, http://links.lww.com/JS9/B985) confirmed the favorable discrimination. The model also had favorable calibration, with the calibration slopes of 1.245 in the external validation cohort 1 (Supplementary Figure 3G, Supplemental Digital Content 13, http://links.lww.com/JS9/B985) and 0.992 in the external validation cohort 2 (Supplementary Figure 3H, Supplemental Digital Content 13, http://links.lww.com/JS9/B985). Furthermore, DCA displayed favorable clinical net benefits in the external validation cohort 1 (Supplementary Figure 3I, Supplemental Digital Content 13, http://links.lww.com/JS9/B985) and in the external validation cohort 2 (Supplementary Figure 3J, Supplemental Digital Content 13, http://links.lww.com/JS9/B985).

### Feature importance

In the entire cohort, the analysis of feature importance revealed that the top five influential factors were ECOG, preoperative ambulatory status, total cholesterol, PT, and albumin for both the training cohort (Supplementary Figure 4A, Supplemental Digital Content 15, http://links.lww.com/JS9/B987) and the validation cohort (Supplementary Figure 4B, Supplemental Digital Content 15, http://links.lww.com/JS9/B987). Moreover, feature importance analysis was conducted for individualized cases using the SHAP method. Supplementary Figures 4C and D (Supplemental Digital Content 15, http://links.lww.com/JS9/B987) highlight a true negative case, where ECOG, preoperative ambulatory status, surgical site, Bilsky score, age, PT, and number of comorbidities were identified as protective factors, while total cholesterol was considered a risk factor. In this case, the most three significant features were ECOG, preoperative ambulatory status, and surgical site. Each feature was assigned a SHAP value, and the total SHAP value in each case represented the cumulative sum of values from all features. For this particular case, the total SHAP value was −5.588, which was lower than the base value of 0.022, indicating a low-risk of experiencing inability to walk. Conversely, Supplementary Figures 4E and F (Supplemental Digital Content 15, http://links.lww.com/JS9/B987) illustrates a true positive case, where the majority of features were identified as risk factors, with the exception of albumin. Additionally, the three most important features in this case were ECOG, preoperative ambulatory status, and PT.

### Comparison of prediction performance in the ensemble model with and without the number of comorbidities

According to the feature importance analysis, the number of comorbidities emerged as the least significant feature. Furthermore, the statistical power analysis revealed that only the number of comorbidities had a statistical power value lower than 0.80 (Supplementary Table 10, Supplemental Digital Content 16, http://links.lww.com/JS9/B988). Consequently, we conducted an investigation and comparison of the predictive performance between the previous model and the new model, excluding the number of comorbidities. We found that the AUC value was still as high as 0.916 (95% CI: 0.863–0.969) (Supplementary Figure 5A, Supplemental Digital Content 17, http://links.lww.com/JS9/B989), and it was slightly greater than the AUC value (0.911) of the previous model including the number of comorbidities. Thus, it demonstrated that the inclusion of the number of comorbidities in the model did not improve the prediction performance. More detailed metrics are summarized in the Supplementary Table 11 (Supplemental Digital Content 18, http://links.lww.com/JS9/B990). Calibration curve of the new model confirmed its favorable calibration, with a calibration slope of 1.132 (Supplementary Figure 5B, Supplemental Digital Content 17, http://links.lww.com/JS9/B989). The discrimination slope was 0.523 (Supplementary Figure 5C, Supplemental Digital Content 17, http://links.lww.com/JS9/B989), and the probability density curve also demonstrated its favorable discrimination (Supplementary Figure 5D, Supplemental Digital Content 17, http://links.lww.com/JS9/B989). DCA showed favorable clinical net benefit (Supplementary Figure 5E, Supplemental Digital Content 17, http://links.lww.com/JS9/B989).

We also tested the prediction performance of the new model in the two external validation cohorts (Supplementary Table 12, Supplemental Digital Content 19, http://links.lww.com/JS9/B991). The results demonstrated that the model exhibited favorable predictive performance in the two external validation cohorts. The AUC values of the new model were 0.880 (95% CI: 0.819–0.940) in the external validation cohort 1 (Supplementary Figure 6A, Supplemental Digital Content 20, http://links.lww.com/JS9/B992) and 0.922 (95% CI: 0.887–0.958) in the external validation cohort 2 (Supplementary Figure 6B, Supplemental Digital Content 20, http://links.lww.com/JS9/B992), still indicating very favorable prediction performance of the new model. The corresponding discrimination slopes were 0.339 (Supplementary Figure 6C, Supplemental Digital Content 20, http://links.lww.com/JS9/B992) and 0.540 (Supplementary Figure 6D, Supplemental Digital Content 20, http://links.lww.com/JS9/B992), respectively, and calibration slopes were 1.323 (Supplementary Figure 6E, Supplemental Digital Content 20, http://links.lww.com/JS9/B992) and 1.035 (Supplementary Figure 6F, Supplemental Digital Content 20, http://links.lww.com/JS9/B992), respectively. The probability density curve demonstrated the favorable discrimination of the new model in the both cohorts (Supplementary Figure 6G, Supplemental Digital Content 20, http://links.lww.com/JS9/B992 and Supplementary Figure 6H, Supplemental Digital Content 20, http://links.lww.com/JS9/B992), and DCA also showed favorable clinical net benefit (Supplementary Figure 6I, Supplemental Digital Content 20, http://links.lww.com/JS9/B992 and Supplementary Figure 6J, Supplemental Digital Content 20, http://links.lww.com/JS9/B992).

### Deployment of the interactive AI platform

**Figure 9:**
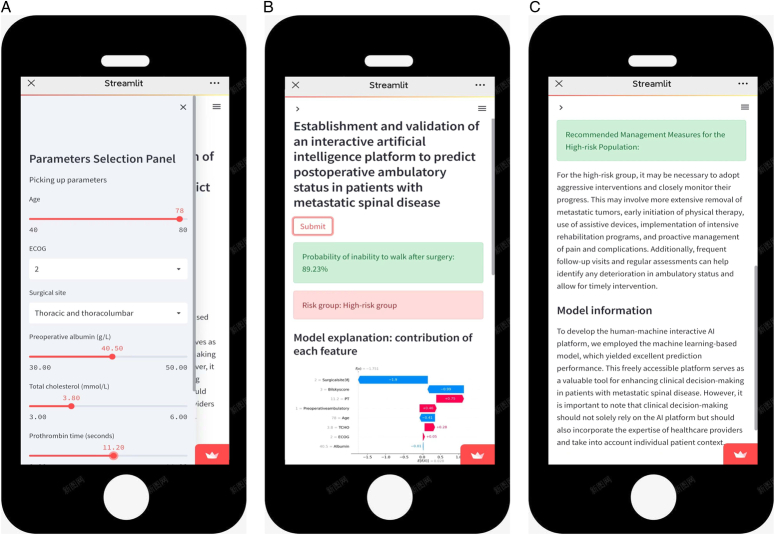
The human-machine interactive artificial intelligence platform. A. Section of inputting data; B. Section of calculating the risk of postoperative inability to walk; C. Section of reporting risk factors and showing model information. Upon accessing the website, users have the option to select relevant parameters for the model feature. Upon clicking the ‘Submit’ button, users will obtain the predicted risk of postoperative inability to walk along with recommended individualized strategy interventions. By utilizing the AI platform and analyzing the contribution of specific features to predictions for each individual case, researchers can identify variables that act as protective factors or risk factors for the individual.

Based on the machine learning-based model, the AI platform has been deployed on the internet at https://postoperativeambulatory-izpdr6gsxxwhitr8fubutd.streamlit.app/. Healthcare professionals can access the AI platform by visiting our provided web link with internet connectivity, using electronic devices such as smartphones, computers, or iPads. Upon visiting the website, users are able to select relevant parameters for the model feature. By clicking the ‘Submit’ button, users will receive the predicted risk of experiencing postoperative inability to walk and recommended individualized strategy interventions (Fig. 9). In addition, by leveraging the AI platform and analyzing the contribution of specific features to predictions for each individual case, researchers can identify variables that serve as protective factors or risk factors for the patient. Furthermore, the AI platform assigns importance rankings to variables on an individualized basis, highlighting the significant risk factors that require greater clinical attention.

In the event that the website is not functioning, users can reactivate the online calculator by clicking ‘Yes, get this app back up!’. By inputting the aforementioned data into the AI platform, users can determine the predicted risk for this specific individual. For instance, a 78-year-old patient, with an ECOG score of 2, treated at the thoracic spine, preoperative albumin level of 40.5 g/l, total cholesterol of 3.8 mmol/l, PT value of 11.2 s, Bilsky score of 3, and preoperative inability to walk, had a predicted risk of inability of 89.23%. Furthermore, the AI platform provides a summary of feature importance for individualized patients and corresponding recommended intervention strategies based on risk stratification. A video of showing how to use the platform is presented in Supplementary File 2 (Supplemental Digital Content 21, http://links.lww.com/JS9/B993).

### A comparison of prediction performance between the AI platform and medical experts

**Figure 10:**
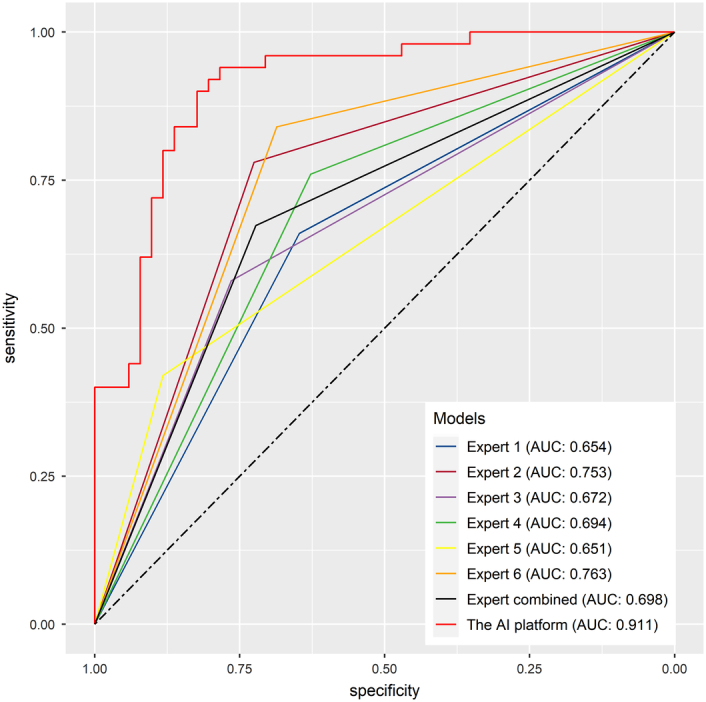
A comparison of prediction performance between the AI platform and medical experts using the area under the curve analysis (*P*<0.001, Delong test).

The AUC values obtained from the six medical experts demonstrated variability, ranging from 0.651 to 0.763 (Fig. 10 and Supplementary Table 13, Supplemental Digital Content 22, http://links.lww.com/JS9/B994). This variability suggested differences in the predictive abilities among the medical experts, with some performing better than others. Notably, the average AUC value of the medical experts was 0.698, which was significantly lower than the AUC value achieved by the AI platform (*P*<0.001, Delong test). The substantial difference in AUC values highlights the superior predictive performance of the AI platform compared to the individual medical experts. The findings of this comparison underscored the potential of the AI platform to surpass the predictive capabilities of individual medical experts. By harnessing the power of advanced algorithms and machine learning techniques, the AI platform can provide more accurate and consistent predictions, leading to more informed clinical decision-making.

## Discussion

### Principal findings

This study aimed to establish and validate an interactive AI platform for predicting postoperative ambulatory status in patients with metastatic spinal disease. The ensemble model performed the best in terms of prediction performance, with other models such as eXGBM, NN, and SVM also showing good results. Key variables influencing the outcome included age, ECOG score, surgical site, preoperative albumin, cholesterol levels, prothrombin time, Bilsky score, and preoperative ambulatory status. We further created an interactive AI platform, providing healthcare professionals with predicted risk assessments and personalized therapeutic recommendations. It also was equipped with good transparency in terms of how certain features contribute to the predictions for each individualized case. Integration of the model into applications and patient-centered explanations of predictions represent opportunities for incorporation into healthcare systems as decision tools in the future.

### Epidemiology of postoperative ambulatory status

The incidence and recovery rates of postoperative ambulation were examined in this study, with 76.82% of patients being able to walk and 98.21% maintained their ambulatory ability in the model derivation cohort. These findings are consistent with previous research^12,14^. For example, Chaichana *et al*.^12^ reported that 78% of patients were able to ambulate postoperatively, and among those who could walk prior to surgery, 89% maintained ambulation. Another study found that 75.4% of patients were able to walk, with 61.9% of previously nonambulatory patients regaining ambulatory status^11^. A meta-analysis including 25 studies showed that 69.7% of patients were able to walk after treatment, with 84.7% maintaining ambulation^14^. It is important to note that ambulatory ability has a significant impact on quality of life and overall survival in patients with spinal metastases^31–33^. Loss of ambulatory ability resulted in an 82% reduction in quality of life among nonambulatory patients^25^ and was associated with a significant decrease in survival outcomes^9^. Hence, it is crucial to prioritize the preservation or restoration of ambulatory status for these patients. Additionally, postoperative ambulatory status is a critical consideration for clinicians when determining the appropriateness of surgical interventions and oncological treatment strategies^8,17^.

### Factors affecting postoperative ambulatory status

Multiple studies have identified various risk factors that influence postoperative ambulatory status^10–14^. For example, a retrospective study revealed that preoperative ambulatory status, presence of pathological vertebral compression fractures at presentation, and utilization of radiation therapy were significant factors associated with postoperative ambulatory status. Preoperative ambulation and postoperative radiotherapy were found to be protective factors for patients with metastatic epidural spinal cord compression^12^. Another study highlighted that presurgical neurological status and prompt surgery for nonambulatory patients were predictors of improved functional outcomes in patients undergoing decompressive surgery for metastatic spinal cord compression^11^. An additional prospective study identified primary tumor type (breast or prostate), ambulatory status grade, bladder function, and prior chemotherapy as significant predictors for increased odds of ambulation after radiotherapy^13^. Notably, a meta-analysis encompassing 25 studies and 4897 patients revealed that ambulatory status before treatment, time interval from symptom onset to treatment, and time of developing motor deficits were significant prognostic factors for post-treatment ambulatory status in patients with metastatic spinal cord compression^14^. Of the included patients, 3173 received radiotherapy^14^. Our study has revealed a correlation between lower preoperative levels of albumin and total cholesterol, as well as higher levels of PT, and the increased likelihood of postoperative inability to walk. Reduced albumin and total cholesterol levels are indicative of malnutrition or underlying health conditions, which may compromise the body’s capacity to heal and recuperate following surgical intervention, thereby impacting ambulatory function. Conversely, elevated PT levels signify impaired blood clotting function, potentially resulting in prolonged recovery and impeding mobility. By identifying the risk factors that influence postoperative ambulatory status, healthcare professionals can better assess and predict the mobility outcomes of patients undergoing surgery. This knowledge can guide treatment decisions, such as the timing of surgery and the use of interventions like radiation therapy, to optimize patient outcomes. Furthermore, by incorporating these factors into predictive models, healthcare professionals can more accurately predict the postoperative ambulatory status of patients.

### Prediction of ambulatory status

Several scores have been developed to predict ambulatory status among patients with metastatic spinal cord compression who underwent radiotherapy. For instance, a retrospective study proposed a scoring system incorporating five features: primary tumor type, time interval from tumor diagnosis, presence of visceral metastases at the time of radiotherapy, motor function prior to radiotherapy, and time of developing motor deficits^34^. In the scoring system, each feature was assigned a score, and the cumulative score was used to estimate the likelihood of post-treatment ambulation, with higher scores indicating higher ambulatory rates. This scoring system was subsequently validated in a prospective study, demonstrating its effectiveness in distinguishing different probabilities of post-treatment ambulatory status^35^. Nonetheless, these scores were primarily designed for patients undergoing radiotherapy, and evaluation metrics such as AUC, accuracy, and sensitivity were not assessed. Therefore, their generalizability to patients receiving decompressive surgery might be very limited. In the present study, it addresses a critical clinical need by developing an AI platform specifically designed to assess the risk of postoperative loss of ambulatory ability in patients with metastatic spinal disease. This fills a gap in current clinical tools, providing healthcare professionals with a valuable resource for making informed therapeutic strategies. Additionally, the study employed a variety of machine learning techniques to develop prediction models. This comprehensive approach allowed for a thorough evaluation of different modeling methods and identified the ensemble machine learning-based model as the optimal one. Furthermore, we rigorously fine-tuned and evaluated the model using cross-validation, splitting the dataset into 10 equal parts to assess its generalization ability. This approach helped to mitigate overfitting and select hyperparameters resulting in the best average performance across all folds. By incorporating extensive hyperparameter ranges, the AI platform effectively captures diverse characteristics and complexities of individual cases, leading to more accurate predictions. It is worth noting that while adding more features to the model could improve prediction performance, an excessive number of features may diminish clinical utility, which is not conducive to the clinical application of our AI platform. Therefore, in our final model, we included eight clinical features as model input features, and the model achieved an AUC value of 0.916, with AUC values of 0.880 in the external validation cohort 1 and 0.922 in the external validation cohort 2.

The study’s methodology, which involved collecting data from multiple medical institutions and using external validation cohorts, provides a strong foundation for assessing the generalizability of the developed AI models. By including patients from different institutions, the study has taken a step toward capturing the variability and diversity present in real-world clinical settings. The results of the external validation demonstrated favorable performance of the ensemble machine learning model across the different cohorts, indicating the potential generalizability of the model in predicting postoperative ambulatory status for patients with metastatic spinal disease. Furthermore, the deployment of an interactive AI platform based on the final machine learning model allows for broader accessibility and utilization of the predictive tool. By making the AI platform available online, researchers and healthcare professionals from various institutions and clinical settings can utilize the predictive model, gaining insights into individual predicted risks and accessing stratified therapeutic recommendations.

### Intervention guidance under the AI model

The AI platform developed in our study has the capability to automatically stratify patients into high and low-risk groups for postoperative ambulatory status after healthcare professionals input patient variable parameters. This functionality streamlines the process for healthcare professionals and enables them to quickly and accurately assess patient risk, leading to more targeted and effective interventions and ultimately improving patient outcomes. In detail, identifying patients at high-risk of inability to walk after surgery allows surgeons to tailor their therapeutic strategies. For the high-risk group, aggressive surgical interventions and close monitoring may be warranted, including considerations such as widely excision of metastatic tumors, earlier initiation of physical therapy, utilization of assistive devices, intensive rehabilitation programs, and proactive management of pain and complications. Additionally, closer follow-up visits and frequent assessments can help detect any deterioration in ambulatory status and allow for timely intervention. Notably, if patients are at a high-risk of postoperative ambulatory impairment, and if their potential benefits from the surgery, such as pain relief, are exceedingly limited, clinical consideration of the appropriateness of surgery for such patients is crucial. In such cases, a careful evaluation is warranted to weigh the potential risks and benefits, ensuring that surgical interventions are aligned with the best interests of the patient’s overall well-being. This study’s interactive AI platform, which accurately predicts postoperative ambulatory status, can aid healthcare professionals in making informed decisions about surgical interventions, further enhancing the patient-centered approach to care, and the AI platform is particularly valuable in cases where the risk of postoperative ambulatory loss is high, as it allows for more tailored and patient-specific treatment recommendations. On the other hand, for the low-risk group, a more conservative approach may be taken. Resources can be allocated more efficiently and interventions can be focused on minimizing complications rather than restoring ambulatory status. This group may require less intensive rehabilitation and follow-up, allowing resources to be redirected to patients who need them more. By stratifying patients into high and low-risk groups, this AI model provides clinicians with valuable information to guide their decision-making and optimize patient outcomes. It can help clinicians allocate resources effectively, provide personalized care plans, and tailor interventions based on individual patient needs. Ultimately, this approach can lead to improved patient experiences, better surgical outcomes, and more efficient healthcare resource utilization.

Prior to a patient’s surgery, we propose the utilization of our AI platform for all patients with metastatic spinal disease. Through the input of essential model parameters, such as the patient’s ECOG, Bilsky score, and preoperative albumin level, and by clicking the ‘Submit’ button, healthcare professionals can not only obtain the patient’s postoperative ambulatory risk and risk stratification but also receive a report detailing the importance ranking of risk variables. Based on the report, researchers can identify variables that act as protective factors or risk factors for postoperative ambulation in the patient. The AI platform also ranks the importance of variables on an individualized basis, highlighting the significant risk factors that should receive more clinical attentions. For instance, if the ranking of risk factors for a patient suggests that comorbidities have a substantial impact on postoperative ambulatory status, clinical focus should be placed on the treatment and management of these comorbidities. This personalized approach allows healthcare professionals to prioritize interventions and treatments based on the specific needs and risks of each patient, optimizing patient outcomes and improving the overall quality of care.

Notably, the DCA curve demonstrated that interventions had greater benefits when the risk probability was lower, which is reasonable because these interventions had lower costs and could be widely implemented. However, in situations where medical resources were limited and only patients with higher risk thresholds could be selected for intervention, the DCA results showed that the model did not benefit in the external cohort 1. This may be due to some bias in the sample distribution, such as a tendency toward predicting positive results. This could be related to the sampling done during the modeling process, which increased the positivity rate from around 20 to 50%. However, in the external validation of cohort 2, the DCA showed that interventions could still be beneficial when medical resources were limited and patients with risk thresholds were selected. Therefore, we claim that interventions can be carried out at low cost and can be effective even when medical resources are limited.

### Limitations

The limitations of the study should be taken into consideration when interpreting the results. Firstly, the AI platform developed in this study is heavily reliant on the availability and quality of input data. However, the complex nature of the model and potential data noise could lead to overfitting issues. Secondly, while the AI platform demonstrated good predictive performance, the dynamic nature of the disease and potential changes in patient status over time may impact the accuracy of the platform. In addition, the developed AI model was primarily designed to predict the short-term ambulatory status after surgery, and thus additional validation and calibration were required for predicting long-term ambulatory prognosis. Thirdly, cross-validation could help to mitigate the risk of overfitting, but it also could result in fewer outcomes per fold, which might impact the reliability of the model’s performance assessment. Fourthly, the integration of additional variables, such as the duration between spinal cord metastasis onset and surgical intervention, holds potential to enhance the predictive capabilities of the AI platform. Fifthly, while these models provide accurate predictions, the complex algorithms used in machine learning techniques can make it challenging to understand and explain the deeper reasoning behind the predictions. Finally, the generalizability of the AI models should be carefully considered. External validation demonstrated favorable performance, but as we only conducted external validation in two hospitals, it is important to continually assess and refine the models as new data becomes available to ensure their applicability across different patient populations and clinical settings, such as nontertiary hospitals and hospitals in other regions. Thus, while the AI platform shows promise in predicting postoperative ambulatory status in patients with metastatic spinal disease, it is important to address extensive validation of the model with a large sample size to enhance its applicability and utility in clinical practice.

## Conclusions

The study successfully develops and validates an interactive AI platform using machine learning for evaluating the risk of postoperative loss of ambulatory ability in patients with metastatic spinal disease. The AI platform allows researchers to predict the risk of postoperative inability to walk and provide tailored therapeutic recommendations. This interactive AI platform has the potential to guide surgical planning and improve patient outcomes.

## Ethical approval

This study was approved by the Ethics Committee of the Peking University First Hospital (Judgement's reference number: 2022-417-001), the Fifth Medical Center of PLA General Hospital (Judgement's reference number: 2019044D), and the Affiliated Hospital of Academy of Military Medical Sciences (Judgement's reference number: 2013-4-49).

## Consent

Written informed consent was obtained from the patient for publication and any accompanying images. A copy of the written consent is available for review by the Editor-in-Chief of this journal on request.

## Sources of funding

Yaosheng Liu received grants support from the Beijing Natural Science Foundation-Haidian Original Innovation Joint Fund (No. L232064).

## Author contribution

All authors contributed to the study design, conducted the data collection and analyses, and drafted the paper. All authors have read and approved the manuscript.

## Conflicts of interest disclosure

The authors declare that they have no conflicts of interest.

## Research registration unique identifying number (UIN)

1. Registry used: ChiCTR: Chinese Clinical Trial Registry.
2. Registration ID: ChiCTR-POC-16008393.
3. Hyperlink to your specific registration: https://www.chictr.org.cn/showproj.html?proj=14138.

## Guarantor

Mingxing Lei, Xuedong Shi, and Yaosheng Liu.

## Data availability statement

The datasets of the current study are available under reasonable request.

## Provenance and peer review

Not commissioned, externally peer-reviewed.

## Supplementary Material

**Figure:**
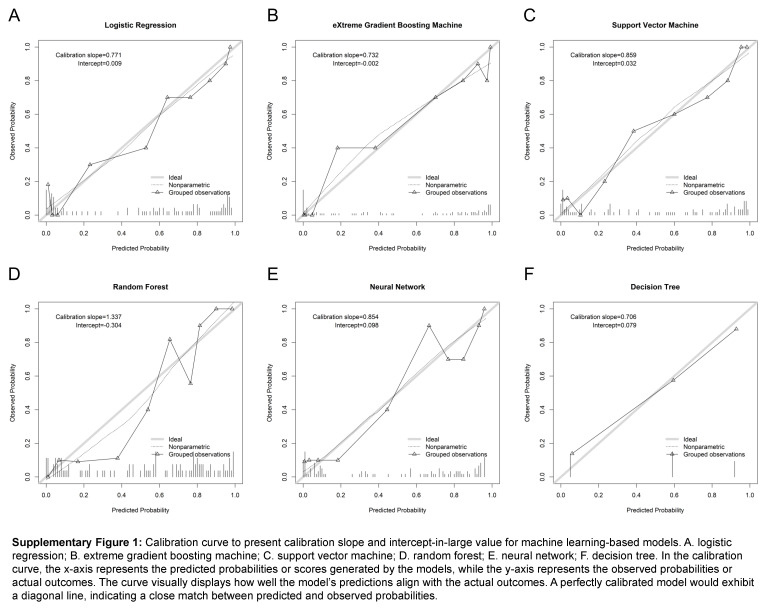


**Figure:**
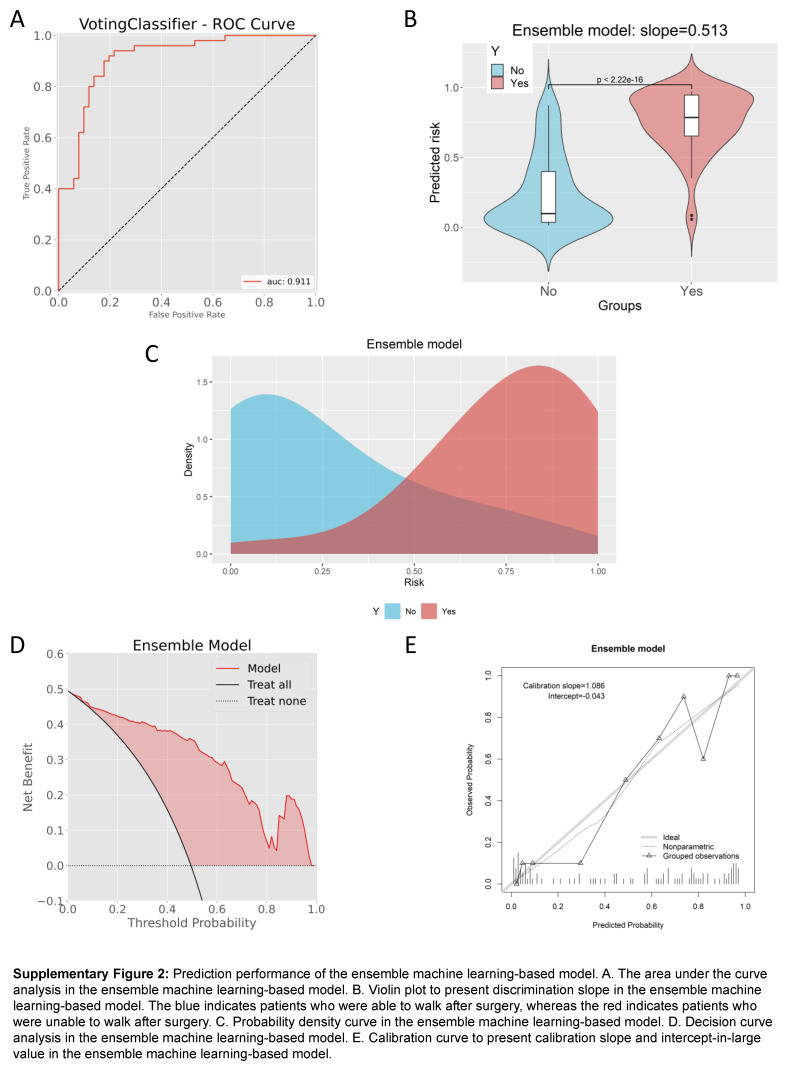


**Figure:**
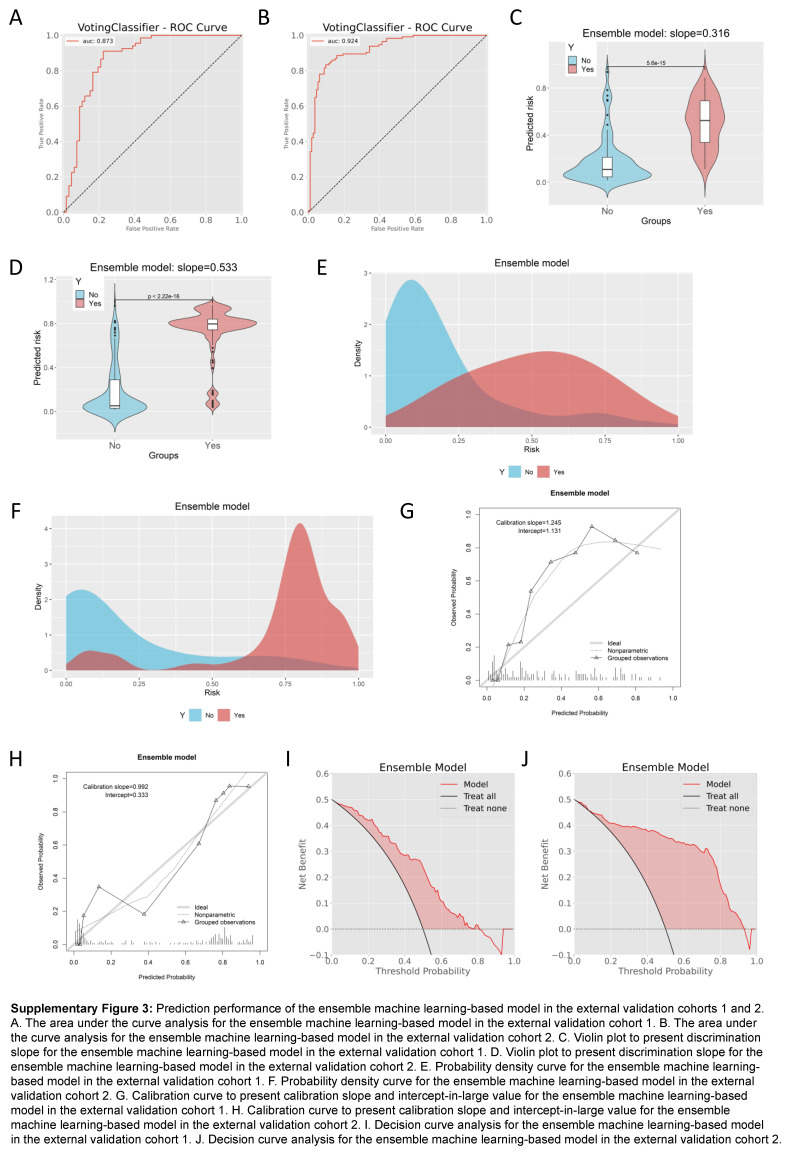


**Figure:**
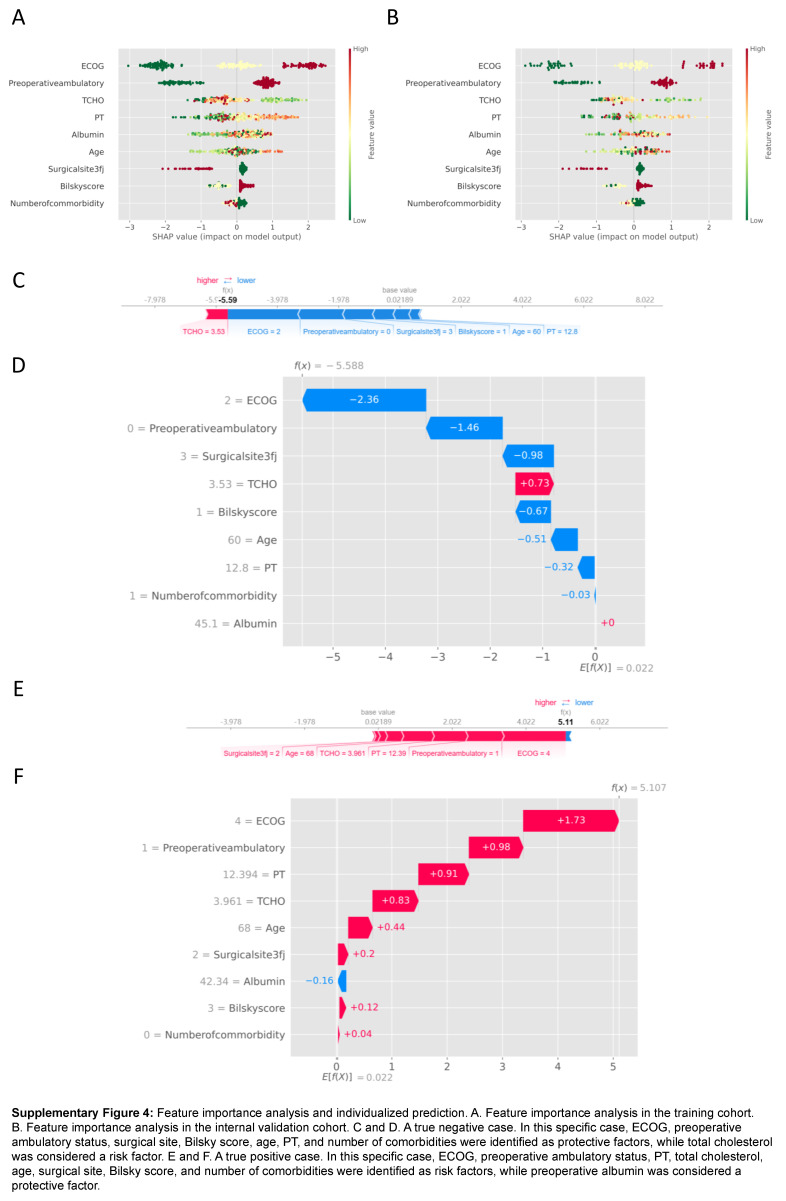


**Figure:**
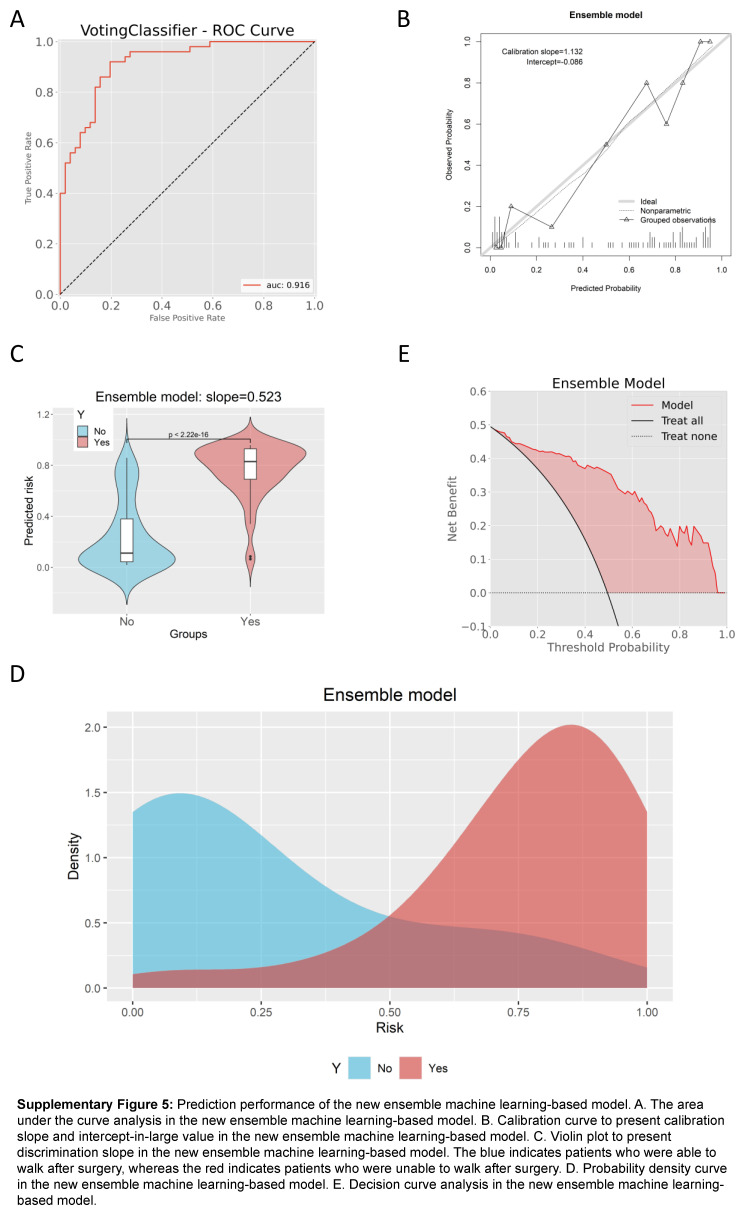


**Figure:**