# Supplementary material for: Establishment and validation of an interactive artificial intelligence platform to predict postoperative ambulatory status for patients with metastatic spinal disease: a multicenter analysis
Source: Int J Surg. 2024 Feb 19;110(5):2738–56. doi: 10.1097/JS9.0000000000001169 (PMC11093492; doi:10.1097/JS9.0000000000001169)
Supplement: Supplementary file 1 [file js9-110-2738-s001.docx]

**Establishment and validation of an interactive artificial intelligence platform to predict postoperative ambulatory status for patients with metastatic spinal disease: a protocol for a multicenter observational study**

**Abstract**

**Purpose:** Identifying patients at high risk of postoperative non-ambulatory status is crucial for surgeons in formulating therapeutic strategies for individuals with metastatic spinal disease. Nevertheless, a clinical tool for assessing postoperative ambulatory status in these patients is currently lacking. The objectives of this study are twofold: firstly, to establish and validate a machine learning-based predictive model for postoperative ambulatory status in patients with metastatic spinal disease; and secondly, to develop an intelligent platform for predicting postoperative ambulatory status in this patient population.

**Methods:** This study will collect patients with metastatic spinal tumors who underwent posterior decompressive surgery at three distinct tertiary medical institutions. We will record patient’s demographic information, comorbidities, information regarding tumor conditions, preoperative ambulatory status, details of oncological therapies, surgical strategies, and laboratory examinations. The principal focus of this study centers on the ambulatory status of patients within one week following their surgical procedure. The interactive AI platform will be established using machine learning techniques to develop prediction models, such as logistic regression (LR), decision tree (DT), random forest (RF), extreme gradient boosting machine (eXGBM), support vector machine (SVM), and neural network (NN). Additionally, to improve the robustness of the model, an ensemble machine learning approach will be implemented using a soft-voting method to combine the results of the aforementioned six algorithms. The interactive AI platform will be further deployed via Streamlit.

**Discussion:** The prevalence of postoperative ambulatory impairment remains high in individuals with metastatic spinal disease, indicating the ongoing relevance and urgency of our current project. Assessing postoperative ambulatory status is not only critical for immediate functional outcomes, but also holds substantial implications for overall prognosis and quality of life for these patients. It is a crucial factor in determining procedural success and overall patient prognosis. Despite its significance, robust clinical tools for predicting postoperative ambulatory status are currently lacking. The existing literature is limited in addressing specific risk factors associated with postoperative ambulation. However, recent advancements in artificial intelligence (AI) and machine learning techniques within the field of spinal metastatic tumors have shown promising results. We hypothesize that integrating AI into the development of predictive models for postoperative ambulatory status will yield favorable prediction performance.

**Registration:** Registered in a national register center.

**1 Introduction**

Metastatic spinal disease refers to the secondary metastases caused by malignant tumors spreading to the spine and/or the extradural space [1]. It is a common and severe complication in advanced malignant tumors. Approximately 50% of patients with metastatic spinal disease eventually experience motor and sensory dysfunction, along with rectal and bladder dysfunction, while 50-68% of patients have already lost their ability to walk at the time of diagnosis. Multidisciplinary sequential treatment can only proceed smoothly when the benefits of surgery outweigh the surgical risks, and when patients can recover and maintain a certain quality of life after a certain period post-surgery [2]. The value and role of surgery can only be demonstrated when these criteria are met.

The ability to ambulate after surgery for metastatic spinal disease is of great importance as it directly impacts the patients' overall quality of life and functional independence [3]. The postoperative ambulatory status serves as a crucial indicator of the patient's immediate recovery and rehabilitation trajectory. For patients with metastatic spinal disease, the capacity to ambulate not only signifies the success of the surgical intervention but also plays a vital role in their mobility, self-care, and daily activities. In addition, the significance of evaluating the postoperative ambulatory status for patients with metastatic spinal disease is further underscored by the scarcity of comprehensive models specifically designed to assess the functional outcomes in this patient population [4]. While numerous studies have focused on developing models to evaluate the survival outcomes of patients with metastatic spinal disease [4-9], the attention given to assessing postoperative ambulation as a critical endpoint is relatively limited [4]. This gap in the literature highlights the necessity of incorporating ambulatory status as a key parameter in prognostic models and treatment decision-making processes, given its substantial impact on the patients' daily functioning and well-being.

Therefore, predicting postoperative ambulatory status in patients with spinal metastases holds significant importance and implications in clinical practice and patient care. It may allow healthcare providers to assess the potential functional outcomes following surgery, aiding in treatment decision-making and patient counseling. By accurately forecasting the postoperative ambulatory status, medical teams can better tailor rehabilitation plans, optimize resource allocation, and provide appropriate support and care for patients undergoing spinal surgery. Furthermore, prognostic predictions contribute to a more comprehensive understanding of the overall treatment effectiveness and patient quality of life, thereby guiding the development of personalized, patient-centered care strategies.

**1.1 Objectives**

(1) Establish and validate a machine learning-based predictive model for postoperative ambulatory status in patients with metastatic spinal disease.

(2) Develop an intelligent platform for predicting postoperative ambulatory status in patients with metastatic spinal disease.

**2 Methods: participants, interventions, and outcomes**

**2.1** **Study settings**

This study will collect patients with metastatic spinal tumors who underwent posterior decompressive surgery at three distinct tertiary medical institutions. The three medical institutions in this study, two of which are located in the northern region of our country and one in the southern region, are all teaching hospitals and are classified as tertiary A-grade hospitals with good reputations. The patient cohort with the largest sample size from one hospital was used as the model derivation cohort, while patients from the other two medical institutions formed the external validation cohorts 1 and 2, respectively. Subsequently, patients in the model derivation cohort were randomly allocated into a training cohort and an internal validation cohort at a ratio of 7:3.

**2.2 Eligibility criteria**

**Inclusion criteria**

The study involved patients who met specific criteria, including having radiographic evidence of metastatic spinal disease and at least one of the following symptoms: progressive local mechanical or radiation pain, impairment of sensory function, lower limb motor function, or sphincter function.

**Exclusion criteria**

Patients receiving conservative treatment, those with primary spinal tumors, metastatic spinal disease caused by leukemia, and intramedullary metastases of spinal metastases were excluded from the study. Additionally, patients who had previously undergone surgery at the site of spinal metastases were also excluded.

Clearly defining inclusive and exclusive criteria for constructing a clinical prediction model offers benefits. It ensured that the study population was well-defined, allowing for a more accurate and specific analysis of the target patient group. Namely, it ensured patient consistency within the study cohort, facilitating the construction of a disease-specific prediction model. To elaborate, by including patients with specific symptoms and radiographic evidence of metastatic spinal disease, we were able to develop a model that was tailored to the unique characteristics and needs of this patient population. Moreover, clear inclusion criteria helped to ensure that the study cohort was homogeneous, minimizing potential confounding variables and enhancing the internal validity of the model. This, in turn, allowed for more accurate predictions and recommendations for patient care within this specific disease context. Additionally, by excluding patients receiving conservative treatment, those with primary spinal tumors, metastatic spinal disease caused by leukemia, and intramedullary metastases of spinal metastases, the study aimed to focus on a specific subset of patients with metastatic spinal disease, thus improving the disease specificity of the prediction model. This targeted approach enhanced the model’s clinical relevance and applicability to the intended patient population.

In detail, leukemia generally presents with unique clinical characteristics and treatment considerations compared to other types of solid tumor metastases [10]. By excluding these patients, the study could focus on a more homogeneous cohort with similar disease manifestations and prognostic factors, which was essential for constructing a specific and relevant prediction model for spinal metastatic disease. Similarly, the exclusion of patients with intramedullary spinal cord metastases was important due to the distinct nature of these cases. Intramedullary metastases often require different treatment approaches and have unique prognostic implications compared to extradural spinal metastases [11, 12]. By focusing specifically on extradural metastatic disease, the study could provide more targeted and applicable insights into this particular subset of spinal metastases.

**2.3 Interventions**

**Intervention description**

The decision to proceed with surgical intervention was predicated on the presence of intractable pain stemming from spinal instability and myelopathy resulting from compression of the spinal cord. The determination of the optimal surgical strategy entailed a collaborative effort involving a neuro-radiologist, spinal tumor surgeon, and oncologist. The surgical management of spinal metastases entailed a multifaceted procedure, encompassing palliative decompression, partial vertebrectomy/en bloc resection of vertebrae, and internal fixation utilizing pedicle screw instrumentation. Under general anesthesia, the patient was positioned in the prone orientation, following which a midline incision was meticulously executed over the affected spinal region, enabling careful dissection of the musculature to expose the posterior spinal elements. Employing a posterior approach, the surgical technique involved either laminectomy or laminotomy to gain access to the spinal cord and nerve roots. Tumor removal was accomplished through a combination of partial vertebrectomy and tumor debulking techniques, with the extent of the vertebrectomy contingent upon tumor involvement. The decision to undertake subtotal or total vertebrectomy was based on comprehensive evaluation of the tumor and the patient's overall health status by the surgical team. Notably, no intradural intervention was necessitated within the scope of this study. Following tumor excision, the resultant space within the vertebral body was filled with bone cement and artificial vertebral bodies to facilitate fusion and stabilization of the spine. Fusion was achieved by addressing the adjacent vertebrae above and below the corpectomy site, involving the insertion of screws into the pedicles of the affected vertebrae, which were interconnected using rods to ensure stability and mitigate the risk of further deformity. The wound was meticulously closed in layers with sutures.

**Criteria for discontinuing**

In the event of an imminent need for safety and protective measures during the research project, the project leader must be promptly notified within 24 hours. Subsequently, the Ethics Committee will be apprised of these measures and the underlying circumstances within 7 days. Should a serious event arise, as outlined by Article 21 of the Ordinance on Human Research (HRO), the research project will be promptly suspended, and the Ethics Committee will be informed within the stipulated 7-day timeframe. A serious event, as defined within this context, encompasses any adverse occurrence where the potential link to the collection of health-related personal data cannot be excluded, and which meets one of the following criteria: necessitates inpatient treatment not originally anticipated in the protocol or extends an existing hospital stay; results in permanent or significant incapacity or disability; or poses a life-threatening risk or culminates in fatality. It is pertinent to emphasize that given the observational nature of the study, treatment-related complications or mortality do not fall within the purview of these regulations. Notably, severe adverse events pertinent to this study are construed as any incidents associated with data collection and processing, including breaches of confidentiality or other comparable occurrences.

**2.4 Outcome**

The principal focus of this study centered on the ambulatory status of patients within one week following their surgical procedure. Ambulatory status was specifically defined as the capability to autonomously take a minimum of two steps with each foot (totaling four steps) [13], even if the use of a cane or walker was required. The capacity to ambulate subsequent to surgery for metastatic spinal disease holds significant importance, as it directly influences the overall quality of life and functional independence of the patients.

**3 Methods: data collection, management, and analysis**

**3.1 Data collection methods**

The data management system is utilized for the collection of outcomes. This system is a validated and internationally used database. Data is extracted from the individual subject file, and a Case Report Form (CRF) is generated for each enrolled patient. The CRF is continually updated to reflect the patient's status during the study. All data entered into the database must also be accessible in the individual subject file, either in the form of worksheets or as notes taken by the investigator or another responsible person designated by the investigator (source documentation). Specific training for the use of the data management system is provided, and plausibility rules are established to enhance data quality. Additionally, data is reviewed and verified prior to completion of data entry. Data quality is further improved through regular data monitoring and planned audits conducted by the CTU Basel. Thirty days after the operation, study patients are interviewed by study nurses to assess postoperative complication rates. In instances where patients are unavailable, treating physicians of the study patients are interviewed.

**3.2 Data management**

The data is securely transferred into the aforementioned password-protected data management system database. The participant, hospital, and physician identification lists are stored within a server of our hospital, separate from the patient data, with access limited to the Principal Investigator (PI) and two Co-PIs. For quality assurance, the Ethics Committee or an independent trial monitor may conduct visits to the research sites at any time. All involved parties maintain strict confidentiality of patient data. The Sponsor-Investigator is responsible for planning and conducting data monitoring. All source data, project-related files, and documents are accessible to the monitor, and any queries are discussed during the monitoring visits.

**3.3 Data monitoring**

The study-specific monitoring is carried out by the orthopaedic department of our hospital. The monitor periodically verifies the implementation of the study and data collection to ensure adherence to the protocol and to ascertain accurate and comprehensive data quality and documentation in the electronic CRF. All source data, project-related files, and documents are made accessible to the monitor, and any inquiries are addressed during the monitoring visits.

**3.4 Data quality control**

This study implemented a comprehensive and meticulous approach to ensure the precision and reliability of the acquired data. The research team underwent extensive training to thoroughly understand the data collection protocols, aiming to minimize potential errors and ensure consistent adherence to standardized guidelines. A rigorous data entry and validation framework was then established, involving a meticulous double-entry verification process by two independent individuals, followed by careful cross-verification to identify any disparities. Furthermore, comprehensive data validation checks were performed to meticulously identify and rectify any inconsistencies or inaccuracies within the collected data. An exhaustive data cleaning procedure was meticulously executed to detect and rectify errors, missing data points, or outliers. This involved a meticulous comparison of the collected data with the source documents, diligently resolving any disparities uncovered. Continuous data monitoring was conducted throughout the study, facilitating the proactive identification of potential issues or trends that could impact data quality. This encompassed periodic audits, meticulous review of data collection forms, and the provision of constructive feedback to the research team to ensure ongoing data quality control. By adhering to these stringent data quality control measures, the study aimed to uphold a standard of excellence in data quality, thereby fortifying the validity and integrity of the research findings.

**3.5 Patient factors**

Patient demographic information, encompassing age, gender, smoking status, and body mass index (BMI), was systematically gathered. Comorbidities, consisting of the number of comorbidities, as well as the presence of coronary disease, diabetes, and hypertension, were documented. Pertinent details regarding tumor conditions, such as the primary tumor type, presence of extra-vertebral bone metastases, viscera metastases, Eastern Cooperative Oncology Group (ECOG) score, Bilsky score, and preoperative ambulatory status, were also diligently recorded. Moreover, information pertaining to oncological therapies, including preoperative chemotherapy, targeted therapy, and endocrinology, was meticulously documented. Surgical strategies, including the surgical process, surgical site, and intraoperative blood transfusion, were also noted. Furthermore, the collection of data involved the inclusion of laboratory examinations, encompassing preoperative albumin, hemoglobin, and prothrombin time (PT).

**3.6 Statistical power analysis**

The power analysis conducted using the PASS statistical software program (Version: 11.0.10) aimed to estimate the minimum sample size required to detect a significant effect size with a desired level of statistical power. This analysis took into consideration factors such as the expected effect size, significance level (alpha), and desired statistical power, typically set at 80% or higher. The analysis was specifically focused on the significant variables identified in the study. A statistical power above 0.80 indicates a high likelihood of detecting true effects in the population being studied. Additionally, a perfect statistical power of 1.000 indicates an extremely high probability of detecting true effects for these specific variables[14-17]. These findings emphasize the robustness of the statistical power for the significant variables, providing confidence in the reliability and validity of the statistical analyses conducted in this study.

**3.7 Machine learning techniques**

A comprehensive analysis was undertaken using a variety of models, including logistic regression (LR) and five machine learning algorithms: Decision Tree (DT), Random Forest (RF), Support Vector Machine (SVM), extreme gradient boosting machine (eXGBM), and K-Nearest Neighbor (KNN). Furthermore, to enhance the resilience of the study’s model, an ensemble machine learning approach was utilized employing a soft-voting method [18, 19]. This technique amalgamated the results of the aforementioned six algorithms, harnessing the power of machine learning to provide comprehensive predictions. Notably, ensemble models often outperform their individual counterparts, showcasing their unparalleled predictive capabilities. All models were provided with the same input features to ensure consistency.

**LR**

LR utilizes a logistic function to distill a linear equation’s output between negative (‘0’) and positive (‘1’) outcomes. Feature weights are unable to be explained as linear regressions because they do not linearly impact the probability. A good approach to assess feature weights in terms of logistic regression depends on treating it as a linear model for the log odds. Users is able to calculate the odds ratio for each feature by obtaining the corresponding weight exponent [20]. Logistic function is depicted below:

$$\sigma(x)=\frac{1}{1+e^{-x}}$$

Among them, the parameters of LR model includes weight $\vec{w}$ and intercept $b$, and thus it can be also presented as $\vec{\theta}=(\vec{w}\cdot\vec{x}+b)$. $\vec{x}$ is the input variable, and LR model is presented as the following equation:

$$f\left( \vec{x};\vec{\theta} \right)=\sigma(\vec{w}\cdot\vec{x}+b)$$

The entire number of parameters of the logistic model is $d+1$, and $d$ indicates the weight of $\vec{w}$ and 1 indicates intercept. The parameters of the logistic regression $\vec{\theta}$ can be fitted using the maximum likelihood estimation. The output probability of the logistic model is:

$$p_{i}=f\left( \vec{x_{i}};\vec{\theta} \right)$$

**DT**

DT is a tree-structured scheme, in which nodes represent the input features and leaves symbolize decision outcomes [21]. It is one of the earliest and most prominent ML techniques, and it has proven to be a valuable tool for extracting meaningful information from measured data and represent a plausible solution for massive data learning tasks [22].

If there are $C$ categories of data in sample dataset $S$. The Gini index is presented as the following equation [23]:

$$Gini\left( S \right)=1=\sum_{i=1}^{c} P_{i}^{2}$$

Where in the study $S$ indicates the training set, $C$ indicates the data class number, and $P_{i}$ indicates the ratio of the sample number in class $i$ to all samples. If the current node corresponds to the training set $S$, and characteristic root $v$ classifies $S$ into $k$ disjoint subsets $S_{1}$, $S_{2}$, $S_{3}$, …, $S_{k}$, that is:

$$S=S_{1}\cup S_{2}\cup S_{3}\cup\ldots S_{k}$$

Thus, the information gain $G\left( S, v \right)$ is presented as follows:

$$G\left( S, v \right)=Gini\left( S \right)-\sum_{i=1}^{k} \frac{\left| S_{i} \right|}{\left| S \right|}Gini(S_{i})$$

**RF**

RF is a combined classifier consisting of many tree-structured classifiers [24], and hence the name is ‘forest’. It is an ensemble learning algorithm, which are aggregated through bootstrap (bagging), and it predicts a sample by combining the predictions from all individual decision trees via averaging or a majority vote [25]. Compared to a single decision tree model, a random forest model exhibits less overfitting, especially when working with a small dataset.

If the $T$ trees of RF are indicated by $\theta_{1}$, $\theta_{2}$,…, $\theta_{T}$, and $w_{i}(x)$ is the average weight. The average RF prediction for the sample $x$ can be presented by weighted average of predictions of all $T$ trees utilizing the weight vector:

$$w_{i}\left( x \right)=\frac{1}{T}\sum_{j=1}^{T} w_{i}(x,\theta_{j})$$

$$\hat{y}\left( x \right)=\sum_{i=1}^{n} w_{i}(x)y(i)$$

**SVM**

SVM is a more recent model of ML techniques. It a supervised learning algorithm, defining a discrimination classifier via a separating hyperplane which is capable of maximizing the margin between negative and positive events [21]. SVMs first translate the input vector into a higher-dimensional feature space and locate the hyperplane that divides the data points into two classes. The instances that are closest to the border have the greatest marginal distance from the decision hyperplane. The generated classifier has a high degree of generalizability and may be used to accurately classify fresh samples. The formula presenting the squared correlation coefficient ($r^{2}$) of SVM is written as [26]:

$$r^{2}=\frac{(\bar{I}\sum_{i=1}^{\bar{I}} f(x_{i})y_{i}-(\sum_{i=1}^{\bar{I}} {f(x_{i})\sum_{i=1}^{\bar{I}} y_{i})}^{2}}{(\bar{I}\sum_{i=1}^{\bar{I}} {f(x_{i})}^{2}-(\sum_{i=1}^{\bar{I}} {f(x_{i}))}^{2})(\bar{I}\sum_{i=1}^{\bar{I}} {y_{i}}^{2}-(\sum_{i=1}^{\bar{I}} {y_{i})}^{2})}$$

**eXGBM**

eXGBM is an ensemble learning method, and it is able to integrate weak learners in a strong one so that the loss function decreases gradually, establishing additional base-learners in an iterative manner, which have a maximal correlation with the negative slope of a cost function [25]. It is widely used due to its effectiveness, and its prediction performance is superior to the logistic regression model [27].

The eXGBM technique utilizes the decision tree as weak learners:

$$T\left( \vec{x};\vec{\theta} \right)=\sum_{j=1}^{J} \gamma_{j}I(\vec{x}{\in R}_{j})$$

Among them, $j$ represents the number of leaves, and the disjoint sections $R_{j}$ determine it$.$ Each region’s values are represented by $\gamma_{j}$. $\vec{\theta}$ represents a collection of parameters of the decision tree, $I(\vec{x}{\in R}_{j})$ is the indicator function for the region $R$ defined as:

$$I\left( \vec{x}{\in R}_{j} \right)=\left\{ \begin{aligned} 1 (\vec{x}{\in R}_{j}) \\ 0 (\vec{x}\notin R_{j}) \end{aligned} \right.$$

The eXGBM model includes $M$ decision trees with parameters $\vec{\theta}=(\vec{\theta_{1}},\ldots,\vec{\theta_{M}})$. Hence, the eXGBM model is shown as follows:

$$g\left( \vec{x};\vec{\theta} \right)=\sum_{m=1}^{M} T(\vec{x};\vec{\theta_{m}})$$

$$f\left( \vec{x};\vec{\theta} \right)=\sigma(g(\vec{x};\vec{\theta}))$$

**KNN**

KNN algorithm is a type of instance-based learning, or lazy learning, where the function is only approximated locally and all computation is deferred until function evaluation [28]. In this algorithm, an object is classified by a majority vote of its neighbors, with the object being assigned to the class most common among its k nearest neighbors (k is a positive integer, typically small). If k = 1, then the object is simply assigned to the class of its nearest neighbor.

The Euclidean distance formula for two points (x1, y1) and (x2, y2) in a two-dimensional space is given by:

Euclidean Distance = $\sqrt{({x2-x1)}^{2}+{(y2-y1)}^{2}}$

In a multidimensional space, the Euclidean distance formula for two points (x1, y1, z1, ..., n1) and (x2, y2, z2, ..., n2) is given by:

Euclidean Distance =$\sqrt{{{(sum}_{i=1})}^{n}{(x_{2i} - x_{1i})}^{2}}$

Where n is the number of dimensions.

**Ensemble**

In order to bolster the robustness of the study's model, an ensemble machine learning approach utilizing a soft-voting method was employed. This technique amalgamated the results of the aforementioned six algorithms, harnessing the power of machine learning to provide comprehensive and accurate predictions.

$$y=argmax\sum_{j=1}^{m} w_{j}p_{ij}$$

The predicted probability of inability to walk was estimated through adding up predicted probabilities $p$ from individual classifiers, where $w_{j}$ is the weight assigned to the $j$th classifier.

**3.8 Modeling process**

A data preprocessing pipeline utilizing scikit-learn was employed to ensure a consistent and reproducible transformation of the data. This pipeline combined multiple preprocessing steps into a single object to enhance the accuracy and reliability of machine learning models. It involved data transformation, feature selection, data splitting, and standardization and normalization. Imbalanced data was addressed using a SMOTETomek resampling strategy, which combines Synthetic Minority Oversampling Technique (SMOTE) and Tomek Links Undersampling [29]. This strategy aimed to generate a new dataset with a larger sample size and a more balanced distribution of data, consequently enhancing statistical power and generalizability of the findings. Additionally, a stratified strategy was employed to maintain consistent proportions of the outcome classes between ambulatory and non-ambulatory patients. Grid and random hyperparameter searches were performed to identify the optimal hyperparameters for each model, with the area under the curve (AUC) used as the optimization metric. During the grid and random hyperparameter searches, 10-fold cross-validation was used to evaluate the performance of different hyperparameter combinations. This technique was employed to assess the generalization ability of a model by splitting the dataset into 10 equal parts, mitigating the risk of overfitting and providing a reliable estimate of the model’s performance. Wide ranges were established for the hyperparameters to accommodate the variability in model performance, allowing the AI platform to capture the diverse characteristics and complexities of each individual case, leading to more accurate predictions. The machine learning algorithms were implemented using Python (version 3.9.7), and hyperparameter tuning was conducted using Python scikit-learn (version 1.2.2).

**3.9 Validation metrics**

To validate the models, both internal and external validation cohorts were utilized, and a diverse range of evaluation metrics were employed. These metrics included the area under the curve (AUC), accuracy, precision, recall, specificity, Brier score, log loss, discrimination slope, calibration slope, and intercept-in-large. The AUC value was derived using 100 bootstraps. Moreover, accuracy, precision, and recall were calculated using a confusion matrix, with the following equation, where TP, TN, FP, and FN represent true positive, true negative, false positive, and false negative, respectively.

Accuracy =$(TP+TN)/(TP+FN+FP+TN)$

Precision = $TP/(TP+FP)$

Recall (Sensitivity) = $TP/(TP+FN)$

Specificity = $1-FP/(TN+FP)$

The Brier score, was calculated using the formula where $N$ represents the total sample, $p_{i}$ represents the predicted risk, and $o_{i}$ represents the actual probability.

$$Brier Score= \frac{1}{N}\sum_{i=1}^{n} {(p_{i}-o_{i})}^{2}$$

The log loss, calculated using the scikit-learn formula, is a metric that evaluates the quality of classification model predictions. It takes into account the number of samples ($N$), the number of classes ($M$), the true labels ($y_{ij}$), and the predicted probabilities ($p_{ij}$).

$$Log Loss=- \frac{1}{N}\sum_{i=1}^{N} \sum_{j=1}^{M} y_{ij}log(p_{ij})$$

The discrimination slope was computed as the mean variance between the predicted probabilities of patients with and without postoperative ambulatory status. The calibration slope and intercept-in-large were derived from the calibration curve. Furthermore, a scoring system was implemented to comprehensively assess the prediction performance of the models, with each metric rated on a scale of 1 to 6, resulting in a scoring system ranging from 0 to 60. Finally, decision curve analysis (DCA) was utilized to evaluate the clinical net benefits for each model.

**4 Ethics and dissemination**

**4.1 Protocol amendments**

Significant revisions to the project structure, protocol, and associated documentation will be presented to the Ethics Committee for review and approval in accordance with HRO Art. 18 prior to implementation, unless urgent action is necessary to safeguard the welfare of the participants.

**4.2 Consent or assent**

In accordance with local hospital protocols, general consent will be obtained upon admission or, in the event of expedited emergency admission during the course of treatment. Specialized study nurses or the responsible study physicians, based on center-specific guidelines, will collect general consent. The study has received approval from the local Ethics Committee.

**4.3 Confidentiality**

Project data is treated with the highest level of confidentiality, and access is restricted to authorized personnel with a legitimate need for the data to fulfill their responsibilities within the research project. Participants are exclusively identified by a unique participant number on the Case Report Form (CRF) and other project-specific documents. Treating institutions and physicians are pseudonymized within the database, and unblinded interinstitutional benchmarking is prohibited. Data containing identifiable information on the individual contributing hospitals or physicians will not be published.

**4.4 Access to data**

Access to the final trial dataset will be limited to the Principal Investigator (PI), two co-PIs, and the dedicated study statistician.

**4.5 Dissemination policy**

The publication of this study, data sharing, or access to raw data is only allowed with explicit permission from the sponsor-investigator and all Co-PIs. The primary trial publication will be conducted under a group authorship, including all contributing hospitals, their Co-PIs, and co-workers. Side projects will be conducted and published based on the specific contributions of each project, with assignments made during regular meetings of the PI and Co-PIs to individual contributing institutions. Currently, participant-level data and statistical code cannot be accessed.

**5 Discussion**

**5.1 Previous studies on postoperative ambulatory status**

Although a majority of patients regain the ability to walk after surgery for metastatic spinal disease, there remains a notable percentage who are unable to ambulate postoperatively. For instance, in the study by Chaichana et al. [30], 78% of patients regained postoperative ambulation, with 89% maintaining ambulation among those who could walk before surgery. However, the study also indicated that approximately 20% of patients were unable to walk after surgery. Another study reported the similar results with 24.9% of patients having no ability to ambulate after surgery [31]. Furthermore, a comprehensive meta-analysis of 25 studies demonstrated that only 69.7% of patients were capable of walking following treatment, while 30.3% were unable to walk after surgery [32].

**5.2 The importance of postoperative walking ability**

Postoperative ambulatory status is a critical consideration for clinicians when determining the appropriateness of surgical interventions and oncological treatment strategies [4, 7]. The ability to walk postoperatively not only influences the immediate functional outcomes but also holds significant implications for the overall prognosis and quality of life of individuals with metastatic spinal disease [33-35]. Moreover, it directly impacts the success of the surgical procedure and the overall prognosis for patients. Moreover, ambulation is closely linked to broader healthcare outcomes such as early mobilization, reduced risk of postoperative complications, and the potential for enhanced psychological well-being [36-38]. Additionally, it is crucial to underscore that ambulatory ability profoundly impacts the quality of life, as evidenced by an 82% reduction in quality of life among non-ambulatory patients [3]. Therefore, evaluating the postoperative ambulatory status is not only essential for understanding the immediate functional outcomes but also holds significant implications for the overall prognosis and quality of life of patients with metastatic spinal disease.

**5.3 Current scoring systems and limitations**

Several scores have been developed to predict posttreatment ambulatory status in patients with metastatic spinal cord compression who undergo radiotherapy. For instance, a retrospective study proposed a scoring system incorporating five features: primary tumor type, time interval from tumor diagnosis, presence of visceral metastases at the time of radiotherapy, motor function prior to radiotherapy, and time of developing motor deficits [39]. In the scoring system, each feature was assigned a score, and the cumulative score was used to estimate the likelihood of posttreatment ambulation, with higher scores indicating higher ambulatory rates. This scoring system was subsequently validated in a prospective study, demonstrating its effectiveness in distinguishing different probabilities of posttreatment ambulatory status [40]. Nonetheless, these scoring systems were primarily designed for patients undergoing radiotherapy, rather than decompressive surgery, and evaluation metrics such as AUC, accuracy, and sensitivity were not assessed. Therefore, their generalizability to patients receiving decompressive surgery would be limited. In the present study, it addresses a critical clinical need by developing an AI platform specifically designed to assess the risk of postoperative loss of ambulatory ability in patients with metastatic spinal disease. This fills a gap in current clinical tools, providing healthcare professionals with a valuable resource for making informed therapeutic strategies.

**5.4 Clinical significance and future applications**

The development of an AI platform specifically designed to assess the risk of postoperative loss of ambulatory ability fills a significant gap in current clinical tools, offering a valuable resource for healthcare professionals to make informed therapeutic strategies. The comprehensive machine learning approach employed in this study holds promising implications for future clinical applications, potentially revolutionizing the prediction and management of postoperative ambulatory status in individuals with metastatic spinal disease. Consequently, we choose to use the machine learning-based model to establish an AI platform to predict postoperative ambulatory status specifically among patients with metastatic spinal disease treated with decompressive surgery.

**6 Competing interests**

None.

**References**

1. Zheng W, Lei M, Liu Y, Lu X, Yu D, Zhang X: **An Algorithm to Stratify the Risk of Postoperative Emotional Distress in Cancer Patients with Advanced Metastatic Spinal Disease**. *Psychol Res Behav Manag* 2020, **13**:721-731.

2. Lawton AJ, Lee KA, Cheville AL, Ferrone ML, Rades D, Balboni TA, Abrahm JL: **Assessment and Management of Patients With Metastatic Spinal Cord Compression: A Multidisciplinary Review**. *J Clin Oncol* 2019, **37**(1):61-71.

3. Schoenfeld AJ, Yeung CM, Tobert DG, Nguyen L, Passias PG, Shin JH, Kang JD, Ferrone ML: **Characterizing Health-Related Quality of Life by Ambulatory Status in Patients with Spinal Metastases**. *Spine* 2022, **47**(2).

4. Cui Y, Lei M, Pan Y, Lin Y, Shi X: **Scoring Algorithms for Predicting Survival Prognosis in Patients With Metastatic Spinal Disease: The Current Status and Future Directions**. *Clin Spine Surg* 2020, **33**(8):296-306.

5. Tabouret E, Cauvin C, Fuentes S, Esterni B, Adetchessi T, Salem N, Madroszyk A, Goncalves A, Casalonga F, Gravis G: **Reassessment of scoring systems and prognostic factors for metastatic spinal cord compression**. *Spine J* 2015, **15**(5):944-950.

6. Yang JJ, Chen CW, Fourman MS, Bongers MER, Karhade AV, Groot OQ, Lin WH, Yen HK, Huang PH, Yang SH *et al*: **International external validation of the SORG machine learning algorithms for predicting 90-day and one-year survival of patients with spine metastases using a Taiwanese cohort**. *Spine J* 2021, **21**(10):1670-1678.

7. Lei M, Li J, Liu Y, Jiang W, Liu S, Zhou S: **Who are the Best Candidates for Decompressive Surgery and Spine Stabilization in Patients With Metastatic Spinal Cord Compression?: A New Scoring System**. *Spine (Phila Pa 1976)* 2016, **41**(18):1469-1476.

8. Tomita K, Kawahara N, Kobayashi T, Yoshida A, Murakami H, Akamaru T: **Surgical strategy for spinal metastases**. *Spine* 2001, **26**(3):298-306.

9. Tokuhashi Y, Matsuzaki H, Oda H, Oshima M, Ryu J: **A revised scoring system for preoperative evaluation of metastatic spine tumor prognosis**. *Spine (Phila Pa 1976)* 2005, **30**(19):2186-2191.

10. Fujikawa T, Kishimoto K, Inoue S, Nishimura A, Tojo R, Uemura S, Nakamura S, Saito A, Kozaki A, Ishida T *et al*: **Epidural Spinal Cord Compression as the Presenting Manifestation of Acute Myeloid Leukemia: A Case Report and Literature Review**. *Intern Med* 2023, **62**(3):453-457.

11. Rades D, Schiff D: **Chapter 15 - Epidural and intramedullary spinal metastasis: clinical features and role of fractionated radiotherapy**. In: *Handbook of Clinical Neurology. Volume 149*, edn. Edited by Schiff D, van den Bent MJ: Elsevier; 2018: 227-238.

12. Hommadi M, Belemlih M, Marnouch E, Maghous A, Zaghba N, Hamidi FZ, Bazzine A, Saghir KA, Elmarjany M, Sifat H *et al*: **Intramedullary spinal cord metastases: Report of three cases and review of the literature**. *Cancer/Radiothérapie* 2021, **25**(2):169-174.

13. Patchell RA, Tibbs PA, Regine WF, Payne R, Saris S, Kryscio RJ, Mohiuddin M, Young B: **Direct decompressive surgical resection in the treatment of spinal cord compression caused by metastatic cancer: a randomised trial**. *Lancet* 2005, **366**(9486):643-648.

14. Chow SC, Shao J, Wang H: **Sample Size Calculations in Clinical Research**: Marcel Dekker. New York; 2003.

15. D'Agostino RB, Chase W, Belanger A: **The Appropriateness of Some Common Procedures for Testing the Equality of Two Independent Binomial Populations**. *The American Statistician* 1988, **42**:198-202.

16. Fleiss JL, Levin B, Paik MC: **Statistical Methods for Rates and Proportions.Third Edition.**: John Wiley & Sons. New York.; 2003.

17. Machin D, Campbell M, Fayers P, and Pinol A: **Sample Size Tables for Clinical Studies, 2nd Edition.**: Blackwell Science. Malden, Mass.; 1997.

18. Long Z, Yi M, Qin Y, Ye Q, Che X, Wang S, Lei M: **Development and validation of an ensemble machine-learning model for predicting early mortality among patients with bone metastases of hepatocellular carcinoma**. *Front Oncol* 2023, **13**:1144039.

19. Nanayakkara S, Fogarty S, Tremeer M, Ross K, Richards B, Bergmeir C, Xu S, Stub D, Smith K, Tacey M *et al*: **Characterising risk of in-hospital mortality following cardiac arrest using machine learning: A retrospective international registry study**. *Plos Medicine* 2018, **15**(11).

20. Garnica-Caparros M, Memmert D: **Understanding gender differences in professional European football through machine learning interpretability and match actions data**. *Sci Rep* 2021, **11**(1):10805.

21. Kourou K, Exarchos TP, Exarchos KP, Karamouzis MV, Fotiadis DI: **Machine learning applications in cancer prognosis and prediction**. *Comput Struct Biotechnol J* 2015, **13**:8-17.

22. Kaminski B, Jakubczyk M, Szufel P: **A framework for sensitivity analysis of decision trees**. *Cent Eur J Oper Res* 2018, **26**(1):135-159.

23. Wang B, He Z, Yi Z, Yuan C, Suo W, Pei S, Li Y, Ma H, Wang H, Xu B *et al*: **Application of a decision tree model in the early identification of severe patients with severe fever with thrombocytopenia syndrome**. *PLoS One* 2021, **16**(7):e0255033.

24. Cutler DR, Edwards TC, Jr., Beard KH, Cutler A, Hess KT, Gibson J, Lawler JJ: **Random forests for classification in ecology**. *Ecology* 2007, **88**(11):2783-2792.

25. Ancuceanu R, Dinu M, Neaga I, Laszlo FG, Boda D: **Development of QSAR machine learning-based models to forecast the effect of substances on malignant melanoma cells**. *Oncol Lett* 2019, **17**(5):4188-4196.

26. Chen HY, Chen JQ, Li JY, Huang HJ, Chen X, Zhang HY, Chen CY: **Deep Learning and Random Forest Approach for Finding the Optimal Traditional Chinese Medicine Formula for Treatment of Alzheimer's Disease**. *J Chem Inf Model* 2019, **59**(4):1605-1623.

27. Seto H, Oyama A, Kitora S, Toki H, Yamamoto R, Kotoku J, Haga A, Shinzawa M, Yamakawa M, Fukui S *et al*: **Gradient boosting decision tree becomes more reliable than logistic regression in predicting probability for diabetes with big data**. *Sci Rep* 2022, **12**(1):15889.

28. Kongsompong S, T EK, Chumnanpuen P: **K-Nearest Neighbor and Random Forest-Based Prediction of Putative Tyrosinase Inhibitory Peptides of Abalone Haliotis diversicolor**. *Molecules* 2021, **26**(12).

29. Zhu C, Xu Z, Gu Y, Zheng S, Sun X, Cao J, Song B, Jin J, Liu Y, Wen X *et al*: **Prediction of post-stroke urinary tract infection risk in immobile patients using machine learning: an observational cohort study**. *J Hosp Infect* 2022, **122**:96-107.

30. Chaichana KL, Woodworth GF, Sciubba DM, McGirt MJ, Witham TJ, Bydon A, Wolinsky JP, Gokaslan Z: **Predictors of ambulatory function after decompressive surgery for metastatic epidural spinal cord compression**. *Neurosurgery* 2008, **62**(3).

31. Hideyuki K, Hiroto K, Yoko H, Seiko K, Seiji O, Tsukasa Y: **Predictors of Postoperative Gain in Ambulatory Function After Decompressive Surgery for Metastatic Spinal Cord Compression**. *Anticancer Research* 2023, **43**(4):1767.

32. Liu YH, Hu YC, Yang XG, Lun DX, Wang F, Yang L, Zhang H, Feng JT, Hua KC: **Prognostic Factors of Ambulatory Status for Patients with Metastatic Spinal Cord Compression: A Systematic Review and Meta-Analysis**. *World Neurosurg* 2018, **116**:e278-e290.

33. Truong VT, Shedid D, Al-Shakfa F, Hattou L, Shen J, Boubez G, Yuh SJ, Wang Z: **Surgical Intervention for Patients With Spinal Metastasis From Lung Cancer: A Retrospective Study of 87 Cases**. *Clin Spine Surg* 2021, **34**(3):E133-E140.

34. Kakutani K, Sakai Y, Zhang Z, Yurube T, Takeoka Y, Kanda Y, Miyazaki K, Ohnishi H, Matsuo T, Ryu M *et al*: **Survival Rate after Palliative Surgery Alone for Symptomatic Spinal Metastases: A Prospective Cohort Study**. *J Clin Med* 2022, **11**(21).

35. da Silva GT, Bergmann A, Santos Thuler LC: **Prognostic factors in patients with metastatic spinal cord compression secondary to lung cancer: a systematic review of the literature**. *Eur Spine J* 2015, **24**(10):2107-2113.

36. Sugita S, Hozumi T, Yamakawa K, Goto T, Kondo T: **Risk factors for surgical site infection after posterior fixation surgery and intraoperative radiotherapy for spinal metastases**. *Eur Spine J* 2016, **25**(4):1034-1038.

37. Gao L, Cao Y, Cao X, Shi X, Lei M, Su X, Liu Y: **Machine learning-based algorithms to predict severe psychological distress among cancer patients with spinal metastatic disease**. *The Spine Journal* 2023, **S1529-9430**(23):00199-00197.

38. Schoenfeld AJ, Leonard DA, Saadat E, Bono CM, Harris MB, Ferrone ML: **Predictors of 30- and 90-Day Survival Following Surgical Intervention for Spinal Metastases: A Prognostic Study Conducted at Four Academic Centers**. *Spine* 2016, **41**(8).

39. Rades D, Rudat V, Veninga T, Stalpers LJA, Basic H, Karstens JH, Hoskin PJ, Schild SE: **A Score Predicting Posttreatment Ambulatory Status in Patients Irradiated for Metastatic Spinal Cord Compression**. *International Journal of Radiation Oncology Biology Physics* 2008, **72**(3):905-908.

40. Rades D, Douglas S, Huttenlocher S, Rudat V, Veninga T, Stalpers LJA, Basic H, Karstens JH, Hoskin PJ, Adamietz IA: **Validation of a score predicting post-treatment ambulatory status after radiotherapy for metastatic spinal cord compression**. *Int J Radiat Oncol Biol Phys* 2011, **79**(5):1503-1506.
