# Supplementary material for: Establishment and validation of an interactive artificial intelligence platform to predict postoperative ambulatory status for patients with metastatic spinal disease: a multicenter analysis
Source: Int J Surg. 2024 Feb 19;110(5):2738–56. doi: 10.1097/JS9.0000000000001169 (PMC11093492; doi:10.1097/JS9.0000000000001169)
Supplement: Supplementary file 3 [file js9-110-2738-s003.docx]

| **Supplementary Table 1.** The full list of comorbidities for all patients in the model derivation cohort. | |
| --- | --- |
| Comorbidities | Patients (n=220) |
| Hypertension | 68 (30.91%) |
| Diabetes | 30 (13.64%) |
| Chronic heart disease | 12 (5.45%) |
| Chronic liver disease | 13 (5.91%) |
| Chronic kidney disease | 3 (1.36%) |
| Cerebrovascular disease | 11 (5.00%) |
| Chronic lung disease, | 4 (1.82%) |
| Gout | 2 (0.91%) |
| Others | 11 (5.00%) |
