# Supplementary material for: Establishment and validation of an interactive artificial intelligence platform to predict postoperative ambulatory status for patients with metastatic spinal disease: a multicenter analysis
Source: Int J Surg. 2024 Feb 19;110(5):2738–56. doi: 10.1097/JS9.0000000000001169 (PMC11093492; doi:10.1097/JS9.0000000000001169)
Supplement: Supplementary file 4 [file js9-110-2738-s004.docx]

| **Supplementary Table 2.** Patient’s clinical characteristics and a comparison of clinical characteristics between patients with and without postoperative walking ability in the model derivation group. | | | | |
| --- | --- | --- | --- | --- |
| Characteristics | Overall | Postoperative ambulatory status | | p |
|  |  | No | Yes |  |
| n | 334 | 167 | 167 |  |
| Age (years, median [IQR]) | 61.00 [54.00, 70.00] | 60.00 [53.00, 67.00] | 63.00 [58.00, 74.50] | <0.001 |
| Number of comorbidities (%) |  |  |  | 0.736 |
| 0 | 177 (53.0) | 92 (55.1) | 85 (50.9) |  |
| 1 | 110 (32.9) | 53 (31.7) | 57 (34.1) |  |
| ≧2 | 47 (14.1) | 22 (13.2) | 25 (15.0) |  |
| ECOG (%) |  |  |  | <0.001 |
| 1 | 3 (0.9) | 3 (1.8) | 0 (0.0) |  |
| 2 | 116 (34.7) | 106 (63.5) | 10 (6.0) |  |
| 3 | 120 (35.9) | 50 (29.9) | 70 (41.9) |  |
| 4 | 95 (28.4) | 8 (4.8) | 87 (52.1) |  |
| Surgical site (%) |  |  |  | <0.001 |
| Cervical and cervical thoracic | 14 (4.2) | 7 (4.2) | 7 (4.2) |  |
| Thoracic and thoracolumbar | 258 (77.2) | 104 (62.3) | 154 (92.2) |  |
| Lumbar and lumbosacral | 62 (18.6) | 56 (33.5) | 6 (3.6) |  |
| Preoperative albumin (g/L, median [IQR]) | 39.65 [36.85, 42.50] | 40.60 [37.35, 43.20] | 38.88 [36.40, 41.81] | 0.006 |
| Total cholesterol (mmol/L, median [IQR]) | 4.31 [3.63, 4.89] | 4.58 [3.88, 5.20] | 4.09 [3.39, 4.67] | <0.001 |
| PT (seconds, median [IQR]) | 11.40 [10.80, 12.10] | 11.20 [10.50, 11.70] | 11.80 [11.20, 12.30] | <0.001 |
| Bilsky score (%) |  |  |  | <0.001 |
| 1 | 29 (8.7) | 27 (16.2) | 2 (1.2) |  |
| 2 | 88 (26.3) | 58 (34.7) | 30 (18.0) |  |
| 3 | 217 (65.0) | 82 (49.1) | 135 (80.8) |  |
| Preoperative ambulatory status (yes/no, %) | 120/214 (35.9/64.1) | 110/57 (65.9/34.1) | 10/157 (6.0/94.0) | <0.001 |
| IQR, Interquartile range; ECOG, Eastern cooperative oncology group; PT, Prothrombin time. | | | | |
