# Supplementary material for: Establishment and validation of an interactive artificial intelligence platform to predict postoperative ambulatory status for patients with metastatic spinal disease: a multicenter analysis
Source: Int J Surg. 2024 Feb 19;110(5):2738–56. doi: 10.1097/JS9.0000000000001169 (PMC11093492; doi:10.1097/JS9.0000000000001169)
Supplement: Supplementary file 5 [file js9-110-2738-s005.docx]

| **Supplementary Table 3.** Patient’s clinical characteristics and a comparison of clinical characteristics between patients in the training group and the internal validation group. | | | | |
| --- | --- | --- | --- | --- |
| Characteristics | Overall | Training cohort | Validation cohort | p |
| n | 334 | 233 | 101 |  |
| Age (years, median [IQR]) | 61.00 [54.00, 70.00] | 61.00 [54.00, 71.00] | 61.00 [54.00, 68.00] | 0.287 |
| Number of comorbidities (%) |  |  |  | 0.344 |
| 0 | 177 (53.0) | 120 (51.5) | 57 (56.4) |  |
| 1 | 110 (32.9) | 76 (32.6) | 34 (33.7) |  |
| ≧2 | 47 (14.1) | 37 (15.9) | 10 (9.9) |  |
| ECOG (%) |  |  |  | 0.193 |
| 1 | 3 (0.9) | 3 (1.3) | 0 (0.0) |  |
| 2 | 116 (34.7) | 84 (36.1) | 32 (31.7) |  |
| 3 | 120 (35.9) | 76 (32.6) | 44 (43.6) |  |
| 4 | 95 (28.4) | 70 (30.0) | 25 (24.8) |  |
| Surgical site (%) |  |  |  | 0.961 |
| Cervical and cervical thoracic | 14 (4.2) | 10 (4.3) | 4 (4.0) |  |
| Thoracic and thoracolumbar | 258 (77.2) | 179 (76.8) | 79 (78.2) |  |
| Lumbar and lumbosacral | 62 (18.6) | 44 (18.9) | 18 (17.8) |  |
| Preoperative albumin (g/L, median [IQR]) | 39.65 [36.85, 42.50] | 39.40 [36.80, 42.50] | 40.10 [36.90, 42.07] | 0.754 |
| Total cholesterol (mmol/L, median [IQR]) | 4.31 [3.63, 4.89] | 4.30 [3.59, 4.90] | 4.34 [3.74, 4.87] | 0.685 |
| PT (seconds, median [IQR]) | 11.40 [10.80, 12.10] | 11.40 [10.80, 12.03] | 11.41 [10.90, 12.28] | 0.160 |
| Bilsky score (%) |  |  |  | 0.467 |
| 1 | 29 (8.7) | 23 (9.9) | 6 (5.9) |  |
| 2 | 88 (26.3) | 62 (26.6) | 26 (25.7) |  |
| 3 | 217 (65.0) | 148 (63.5) | 69 (68.3) |  |
| Preoperative ambulatory status (yes/no, %) | 120/214 (35.9/64.1) | 84/149 (36.1/63.9) | 36/65 (35.6/64.4) | 1.000 |
| Postoperative ambulatory status (yes/no, %) | 167/167 (50.0/50.0) | 116/117 (49.8/50.2) | 51/50 (50.5/49.5) | 1.000 |
| IQR, Interquartile range; ECOG, Eastern cooperative oncology group; PT, Prothrombin time. | | | | |
