# Supplementary material for: Establishment and validation of an interactive artificial intelligence platform to predict postoperative ambulatory status for patients with metastatic spinal disease: a multicenter analysis
Source: Int J Surg. 2024 Feb 19;110(5):2738–56. doi: 10.1097/JS9.0000000000001169 (PMC11093492; doi:10.1097/JS9.0000000000001169)
Supplement: Supplementary file 6 [file js9-110-2738-s006.docx]

| **Supplementary Table 4**. Hyperparameters of the models. | |
| --- | --- |
| Models | Hyperparameters |
| Logistic regression | LogisticRegression(C=10, random_state=42) |
| eXGBoosting Machine | XGBClassifier(max_features='auto', min_samples_leaf=3, min_samples_split=89, random_state=42) |
| Neural network | MLPClassifier(hidden_layer_sizes=100, random_state=42) |
| Random forest | RandomForestClassifier(max_depth=78, max_features='auto', min_samples_leaf=2, min_samples_split=6, n_estimators=26, random_state=42) |
| Support vector machine | SVC(C=0.08048113035416485, gamma=0.25131854655259606, probability=True) |
| Decision Tree | DecisionTreeClassifier(max_depth=22, max_features='auto', min_samples_leaf=3, min_samples_split=89, random_state=42) |
