# Supplementary material for: Establishment and validation of an interactive artificial intelligence platform to predict postoperative ambulatory status for patients with metastatic spinal disease: a multicenter analysis
Source: Int J Surg. 2024 Feb 19;110(5):2738–56. doi: 10.1097/JS9.0000000000001169 (PMC11093492; doi:10.1097/JS9.0000000000001169)
Supplement: Supplementary file 7 [file js9-110-2738-s009.docx]

| **Supplementary Table 5.** Patient’s clinical characteristics and a comparison of clinical characteristics between patients with and without postoperative walking ability in the external validation cohort 1. | | | | |
| --- | --- | --- | --- | --- |
| Characteristics | Overall | Postoperative ambulatory status | | p |
|  |  | No | Yes |  |
| n | 89 | 70 | 19 |  |
| Age (years, median [IQR]) | 58.00 [51.00, 64.00] | 58.00 [51.25, 63.00] | 56.00 [49.00, 65.50] | 0.849 |
| Number of comorbidities (%) |  |  |  | 0.441 |
| 0 | 41 (46.1) | 34 (48.6) | 7 (36.8) |  |
| 1 | 29 (32.6) | 23 (32.9) | 6 (31.6) |  |
| ≧2 | 19 (21.3) | 13 (18.6) | 6 (31.6) |  |
| ECOG (%) |  |  |  | <0.001 |
| 1 | 5 (5.6) | 4 (5.7) | 1 (5.3) |  |
| 2 | 37 (41.6) | 36 (51.4) | 1 (5.3) |  |
| 3 | 29 (32.6) | 24 (34.3) | 5 (26.3) |  |
| 4 | 18 (20.2) | 6 (8.6) | 12 (63.2) |  |
| Surgical site (%) |  |  |  | 0.526 |
| Cervical and cervical thoracic | 11 (12.4) | 8 (11.4) | 3 (15.8) |  |
| Thoracic and thoracolumbar | 43 (48.3) | 36 (51.4) | 7 (36.8) |  |
| Lumbar and lumbosacral | 35 (39.3) | 26 (37.1) | 9 (47.4) |  |
| Preoperative albumin (g/L, median [IQR]) | 37.40 [35.50, 40.10] | 37.20 [35.50, 40.00] | 37.80 [35.25, 40.50] | 0.798 |
| Total cholesterol (mmol/L, median [IQR]) | 4.60 [3.83, 5.15] | 4.68 [3.96, 5.21] | 4.26 [3.66, 4.86] | 0.154 |
| PT (seconds, median [IQR]) | 10.90 [10.20, 11.70] | 10.75 [10.22, 11.55] | 11.10 [10.30, 12.00] | 0.518 |
| Bilsky score (%) |  |  |  | 0.006 |
| 1 | 3 (3.4) | 3 (4.3) | 0 (0.0) |  |
| 2 | 40 (44.9) | 37 (52.9) | 3 (15.8) |  |
| 3 | 46 (51.7) | 30 (42.9) | 16 (84.2) |  |
| Preoperative ambulatory status (yes/no, %) | 58/31 (65.2/34.8) | 51/19 (72.9/27.1) | 7/12 (36.8/63.2) | 0.008 |
| IQR, Interquartile range; ECOG, Eastern cooperative oncology group; PT, Prothrombin time. | | | | |
