# Supplementary material for: Establishment and validation of an interactive artificial intelligence platform to predict postoperative ambulatory status for patients with metastatic spinal disease: a multicenter analysis
Source: Int J Surg. 2024 Feb 19;110(5):2738–56. doi: 10.1097/JS9.0000000000001169 (PMC11093492; doi:10.1097/JS9.0000000000001169)
Supplement: Supplementary file 8 [file js9-110-2738-s010.docx]

| **Supplementary Table 6.** Patient’s clinical characteristics and a comparison of clinical characteristics between patients with and without postoperative walking ability in the external validation cohort 2. | | | | |
| --- | --- | --- | --- | --- |
| Characteristics | Overall | Postoperative ambulatory status | | p |
|  |  | No | Yes |  |
| n | 146 | 116 | 30 |  |
| Age (years, median [IQR]) | 61.00 [53.25, 69.00] | 59.50 [52.00, 67.50] | 64.00 [60.25, 73.00] | 0.005 |
| Number of comorbidities (%) |  |  |  | 0.778 |
| 0 | 72 (49.3) | 56 (48.3) | 16 (53.3) |  |
| 1 | 54 (37.0) | 43 (37.1) | 11 (36.7) |  |
| ≧2 | 20 (13.7) | 17 (14.7) | 3 (10.0) |  |
| ECOG (%) |  |  |  | <0.001 |
| 1 | 3 (2.1) | 3 (2.6) | 0 (0.0) |  |
| 2 | 77 (52.7) | 75 (64.7) | 2 (6.7) |  |
| 3 | 46 (31.5) | 32 (27.6) | 14 (46.7) |  |
| 4 | 20 (13.7) | 6 (5.2) | 14 (46.7) |  |
| Surgical site (%) |  |  |  | <0.001 |
| Cervical and cervical thoracic | 6 (4.1) | 5 (4.3) | 1 (3.3) |  |
| Thoracic and thoracolumbar | 99 (67.8) | 70 (60.3) | 29 (96.7) |  |
| Lumbar and lumbosacral | 41 (28.1) | 41 (35.3) | 0 (0.0) |  |
| Preoperative albumin (g/L, median [IQR]) | 40.35 [37.30, 43.00] | 40.55 [37.30, 43.20] | 39.55 [37.15, 42.38] | 0.697 |
| Total cholesterol (mmol/L, median [IQR]) | 4.44 [3.82, 5.16] | 4.46 [3.83, 5.35] | 4.39 [3.66, 4.79] | 0.394 |
| PT (seconds, median [IQR]) | 11.10 [10.43, 11.97] | 11.10 [10.40, 12.00] | 11.40 [10.75, 11.80] | 0.607 |
| Bilsky score (%) |  |  |  | 0.005 |
| 1 | 22 (15.1) | 21 (18.1) | 1 (3.3) |  |
| 2 | 45 (30.8) | 40 (34.5) | 5 (16.7) |  |
| 3 | 79 (54.1) | 55 (47.4) | 24 (80.0) |  |
| Preoperative ambulatory status (yes/no, %) | 80/66 (54.8/45.2) | 77/39 (66.4/33.6) | 3/27 (10.0/90.0) | <0.001 |
| IQR, Interquartile range; ECOG, Eastern cooperative oncology group; PT, Prothrombin time. | | | | |
