# Supplementary material for: Establishment and validation of an interactive artificial intelligence platform to predict postoperative ambulatory status for patients with metastatic spinal disease: a multicenter analysis
Source: Int J Surg. 2024 Feb 19;110(5):2738–56. doi: 10.1097/JS9.0000000000001169 (PMC11093492; doi:10.1097/JS9.0000000000001169)
Supplement: Supplementary file 9 [file js9-110-2738-s011.docx]

| **Supplementary Table 7.** Patient’s clinical characteristics and a comparison of clinical characteristics between patients with and without postoperative walking ability in the external validation cohort 1. | | | | |
| --- | --- | --- | --- | --- |
| Characteristics | Overall | Postoperative ambulatory status | | p |
|  |  | No | Yes |  |
| n | 134 | 67 | 67 |  |
| Age (years, median [IQR]) | 55.00 [49.00, 63.00] | 58.00 [51.50, 63.00] | 52.00 [49.00, 62.50] | 0.129 |
| Number of comorbidities (%) |  |  |  | 0.728 |
| 0 | 68 (50.7) | 32 (47.8) | 36 (53.7) |  |
| 1 | 40 (29.9) | 22 (32.8) | 18 (26.9) |  |
| ≧2 | 26 (19.4) | 13 (19.4) | 13 (19.4) |  |
| ECOG (%) |  |  |  | <0.001 |
| 1 | 5 (3.7) | 4 (6.0) | 1 (1.5) |  |
| 2 | 40 (29.9) | 35 (52.2) | 5 (7.5) |  |
| 3 | 49 (36.6) | 23 (34.3) | 26 (38.8) |  |
| 4 | 40 (29.9) | 5 (7.5) | 35 (52.2) |  |
| Surgical site (%) |  |  |  | 0.039 |
| Cervical and cervical thoracic | 24 (17.9) | 8 (11.9) | 16 (23.9) |  |
| Thoracic and thoracolumbar | 70 (52.2) | 33 (49.3) | 37 (55.2) |  |
| Lumbar and lumbosacral | 40 (29.9) | 26 (38.8) | 14 (20.9) |  |
| Preoperative albumin (g/L, median [IQR]) | 37.22 [35.50, 39.91] | 37.00 [35.45, 40.00] | 37.32 [35.56, 39.10] | 0.840 |
| Total cholesterol (mmol/L, median [IQR]) | 4.71 [4.01, 5.13] | 4.68 [4.00, 5.23] | 4.71 [4.06, 5.04] | 0.800 |
| PT (seconds, median [IQR]) | 10.99 [10.19, 11.88] | 10.80 [10.20, 11.50] | 11.10 [9.98, 12.19] | 0.629 |
| Bilsky score (%) |  |  |  | 0.041 |
| 1 | 3 (2.2) | 3 (4.5) | 0 (0.0) |  |
| 2 | 63 (47.0) | 36 (53.7) | 27 (40.3) |  |
| 3 | 68 (50.7) | 28 (41.8) | 40 (59.7) |  |
| Preoperative ambulatory status (yes/no, %) | 80/54 (59.7/40.3) | 48/19 (71.6/28.4) | 32/35 (47.8/52.2) | 0.008 |
| IQR, Interquartile range; ECOG, Eastern cooperative oncology group; PT, Prothrombin time. | | | | |
