# Supplementary material for: Establishment and validation of an interactive artificial intelligence platform to predict postoperative ambulatory status for patients with metastatic spinal disease: a multicenter analysis
Source: Int J Surg. 2024 Feb 19;110(5):2738–56. doi: 10.1097/JS9.0000000000001169 (PMC11093492; doi:10.1097/JS9.0000000000001169)
Supplement: Supplementary file 10 [file js9-110-2738-s012.docx]

| **Supplementary Table 8.** Patient’s clinical characteristics and a comparison of clinical characteristics between patients with and without postoperative walking ability in the external validation cohort 2. | | | | |
| --- | --- | --- | --- | --- |
| Characteristics | Overall | Postoperative ambulatory status | | p |
|  |  | No | Yes |  |
| n | 228 | 114 | 114 |  |
| Age (years, median [IQR]) | 63.00 [58.00, 73.00] | 59.50 [52.00, 68.50] | 64.00 [61.00, 77.00] | <0.001 |
| Number of comorbidities (%) |  |  |  | 0.001 |
| 0 | 128 (56.1) | 54 (47.4) | 74 (64.9) |  |
| 1 | 80 (35.1) | 43 (37.7) | 37 (32.5) |  |
| ≧2 | 20 (8.8) | 17 (14.9) | 3 (2.6) |  |
| ECOG (%) |  |  |  | <0.001 |
| 1 | 3 (1.3) | 3 (2.6) | 0 (0.0) |  |
| 2 | 82 (36.0) | 75 (65.8) | 7 (6.1) |  |
| 3 | 111 (48.7) | 31 (27.2) | 80 (70.2) |  |
| 4 | 32 (14.0) | 5 (4.4) | 27 (23.7) |  |
| Surgical site (%) |  |  |  | <0.001 |
| Cervical and cervical thoracic | 8 (3.5) | 5 (4.4) | 3 (2.6) |  |
| Thoracic and thoracolumbar | 179 (78.5) | 68 (59.6) | 111 (97.4) |  |
| Lumbar and lumbosacral | 41 (18.0) | 41 (36.0) | 0 (0.0) |  |
| Preoperative albumin (g/L, median [IQR]) | 40.10 [37.12, 42.70] | 40.55 [37.30, 43.20] | 39.16 [36.92, 42.40] | 0.289 |
| Total cholesterol (mmol/L, median [IQR]) | 4.41 [3.88, 4.85] | 4.46 [3.82, 5.42] | 4.37 [3.93, 4.72] | 0.142 |
| PT (seconds, median [IQR]) | 11.27 [10.60, 11.86] | 11.10 [10.40, 12.00] | 11.37 [10.90, 11.80] | 0.159 |
| Bilsky score (%) |  |  |  | 0.001 |
| 1 | 25 (11.0) | 21 (18.4) | 4 (3.5) |  |
| 2 | 86 (37.7) | 40 (35.1) | 46 (40.4) |  |
| 3 | 117 (51.3) | 53 (46.5) | 64 (56.1) |  |
| Preoperative ambulatory status (yes/no, %) | 89/139 (39.0/61.0) | 77/37 (67.5/32.5) | 12/102 (10.5/89.5) | <0.001 |
| IQR, Interquartile range; ECOG, Eastern cooperative oncology group; PT, Prothrombin time. | | | | |
