# Supplementary material for: Establishment and validation of an interactive artificial intelligence platform to predict postoperative ambulatory status for patients with metastatic spinal disease: a multicenter analysis
Source: Int J Surg. 2024 Feb 19;110(5):2738–56. doi: 10.1097/JS9.0000000000001169 (PMC11093492; doi:10.1097/JS9.0000000000001169)
Supplement: Supplementary file 11 [file js9-110-2738-s014.docx]

| **Supplementary Table 9.** Prediction performance in the external validation cohort for the ensemble model with the number of comorbidities. | | |
| --- | --- | --- |
| Metrics | External validation | |
|  | Cohort 1 | Cohort 2 |
| Accuracy | 0.739 | 0.855 |
| Precise | 0.864 | 0.846 |
| Recall | 0.567 | 0.868 |
| Specificity | 0.910 | 0.842 |
| AUC (95% CI) | 0.873 (0.809-0.936) | 0.924 (0.890-0.959) |
| Brier score | 0.182 | 0.114 |
| Log loss | 0.531 | 0.380 |
| Discrimination slope | 0.316 | 0.533 |
| Intercept-in-large value | 1.131 | 0.333 |
| Calibration slope | 1.245 | 0.992 |
| AUC, area under the curve; CI, confident interval. | | |
