# Supplementary material for: Establishment and validation of an interactive artificial intelligence platform to predict postoperative ambulatory status for patients with metastatic spinal disease: a multicenter analysis
Source: Int J Surg. 2024 Feb 19;110(5):2738–56. doi: 10.1097/JS9.0000000000001169 (PMC11093492; doi:10.1097/JS9.0000000000001169)
Supplement: Supplementary file 12 [file js9-110-2738-s016.docx]

| **Supplementary Table 10.** Statistical power analysis for significant variables between patients with and without postoperative walking ability. | | | | | |
| --- | --- | --- | --- | --- | --- |
| Characteristics | Overall | Postoperative ambulatory status | | p | Statistical power |
|  |  | Yes | No |  |  |
| n | 220 | 169 | 51 |  |  |
| Age (years, median [IQR]) | 60.00 [53.00, 68.00] | 60.00 [53.00, 67.00] | 63.00 [56.00, 74.00] | 0.041 | 0.819 |
| Number of comorbidities (%) |  |  |  | 0.050 | 0.667 |
| 0 | 116 (52.7) | 93 (55.0) | 23 (45.1) |  |  |
| 1 | 68 (30.9) | 54 (32.0) | 14 (27.5) |  |  |
| ≧2 | 36 (16.4) | 22 (13.0) | 14 (27.5) |  |  |
| ECOG (%) |  |  |  | <0.001 | 1.000 |
| 1 | 3 (1.4) | 3 (1.8) | 0 (0.0) |  |  |
| 2 | 108 (49.1) | 106 (62.7) | 2 (3.9) |  |  |
| 3 | 67 (30.5) | 51 (30.2) | 16 (31.4) |  |  |
| 4 | 42 (19.1) | 9 (5.3) | 33 (64.7) |  |  |
| Surgical site (%) |  |  |  | 0.011 | 0.894 |
| Cervical and cervical thoracic | 9 (4.1) | 7 (4.1) | 2 (3.9) |  |  |
| Thoracic and thoracolumbar | 149 (67.7) | 106 (62.7) | 43 (84.3) |  |  |
| Lumbar and lumbosacral | 62 (28.2) | 56 (33.1) | 6 (11.8) |  |  |
| Preoperative albumin (g/L, median [IQR]) | 40.10 [37.20, 42.60] | 40.60 [37.50, 43.20] | 38.90 [36.75, 41.50] | 0.034 | 0.810 |
| Total cholesterol (mmol/L, median [IQR]) | 4.42 [3.71, 5.10] | 4.58 [3.83, 5.19] | 4.08 [3.50, 4.78] | 0.004 | 0.847 |
| PT (seconds, median [IQR]) | 11.29 [10.60, 11.90] | 11.20 [10.50, 11.70] | 11.80 [11.10, 12.50] | 0.001 | 0.871 |
| Bilsky score (%) |  |  |  | <0.001 | 1.000 |
| 1 | 28 (12.7) | 27 (16.0) | 1 (2.0) |  |  |
| 2 | 63 (28.6) | 58 (34.3) | 5 (9.8) |  |  |
| 3 | 129 (58.6) | 84 (49.7) | 45 (88.2) |  |  |
| Preoperative ambulatory status (yes/no, %) | 112/108 (50.9/49.1) | 110/59 (65.1/34.9) | 2/49 (3.9/96.1) | <0.001 | 1.000 |
| IQR, Interquartile range; ECOG, Eastern cooperative oncology group; PT, Prothrombin time. | | | | | |
