# Supplementary material for: Establishment and validation of an interactive artificial intelligence platform to predict postoperative ambulatory status for patients with metastatic spinal disease: a multicenter analysis
Source: Int J Surg. 2024 Feb 19;110(5):2738–56. doi: 10.1097/JS9.0000000000001169 (PMC11093492; doi:10.1097/JS9.0000000000001169)
Supplement: Supplementary file 13 [file js9-110-2738-s018.docx]

| **Supplementary Table 11.** Prediction performance of the ensemble model before and after excluding the number of commorbidities. | | |
| --- | --- | --- |
| Metrics | Ensemble model | |
|  | With the number of commorbidities | Without the number of commorbidities |
| Accuracy | 0.861 | 0.861 |
| Precise | 0.833 | 0.821 |
| Recall | 0.900 | 0.920 |
| Specificity | 0.824 | 0.804 |
| AUC (95% CI) | 0.911 (0.854-0.968) | 0.916 (0.863-0.969) |
| Brier score | 0.118 | 0.114 |
| Log loss | 0.375 | 0.365 |
| Discrimination slope | 0.513 | 0.523 |
| Intercept-in-large value | -0.043 | -0.086 |
| Calibration slope | 1.086 | 1.132 |
| AUC, area under the curve; CI, confident interval. | | |
