# Supplementary material for: Establishment and validation of an interactive artificial intelligence platform to predict postoperative ambulatory status for patients with metastatic spinal disease: a multicenter analysis
Source: Int J Surg. 2024 Feb 19;110(5):2738–56. doi: 10.1097/JS9.0000000000001169 (PMC11093492; doi:10.1097/JS9.0000000000001169)
Supplement: Supplementary file 14 [file js9-110-2738-s019.docx]

| **Supplementary Table 12.** Prediction performance in the external validation cohort for the ensemble model excluding the number of comorbidities. | | |
| --- | --- | --- |
| Metrics | External validation | |
|  | Cohort 1 | Cohort 2 |
| Accuracy | 0.746 | 0.860 |
| Precise | 0.851 | 0.847 |
| Recall | 0.597 | 0.877 |
| Specificity | 0.900 | 0.842 |
| AUC (95% CI) | 0.880 (0.819-0.940) | 0.922 (0.887-0.958) |
| Brier score | 0.168 | 0.111 |
| Log loss | 0.500 | 0.371 |
| Discrimination slope | 0.339 | 0.540 |
| Intercept-in-large value | 1.063 | 0.281 |
| Calibration slope | 1.323 | 1.035 |
| AUC, area under the curve; CI, confident interval. | | |
