# Supplementary material for: Establishment and validation of an interactive artificial intelligence platform to predict postoperative ambulatory status for patients with metastatic spinal disease: a multicenter analysis
Source: Int J Surg. 2024 Feb 19;110(5):2738–56. doi: 10.1097/JS9.0000000000001169 (PMC11093492; doi:10.1097/JS9.0000000000001169)
Supplement: Supplementary file 16 [file js9-110-2738-s022.docx]

| **Supplementary Table 13.** Prediction performance of medical experts in assessing postoperative ambulatory status among patients with metastatic spinal disease. | | | | | | | |
| --- | --- | --- | --- | --- | --- | --- | --- |
| **Metrics** | **Doctors** | | | | | | |
|  | 1 | 2 | 3 | 4 | 5 | 6 | Average |
| AUC | 0.654 | 0.753 | 0.672 | 0.694 | 0.651 | 0.763 | 0.698 |
| Specificity | 0.647 | 0.725 | 0.765 | 0.627 | 0.882 | 0.686 | 0.722 |
| Sensitivity | 0.660 | 0.780 | 0.580 | 0.760 | 0.420 | 0.840 | 0.673 |
| Accuracy | 0.653 | 0.752 | 0.673 | 0.693 | 0.653 | 0.762 | 0.698 |
| Precision | 0.647 | 0.736 | 0.707 | 0.667 | 0.778 | 0.724 | 0.704 |
| AUC, area under the curve. | | | | | | | |
